# In Vitro Analysis and Dynamic Modeling of SARS-CoV-2 Infection Inhibition by Sigma-1 Receptor Antagonist PB28

**DOI:** 10.1007/s11538-026-01642-2

**Published:** 2026-05-23

**Authors:** Bartek Lisowski, Veronica V. Rezelj, Marco Vignuzzi, Carmen Abate, Veronika Bernhauerová

**Affiliations:** 1 Chair of Pharmaceutical Technology and Biopharmaceutics, Faculty of Pharmacy, Jagiellonian University Medical College, Kraków, Poland; 2 Institut Pasteur, Viral Populations and Pathogenesis Unit, Department of Virology, Paris, F-75015 France; 3 A*STAR Infectious Diseases Labs (A*STAR ID Labs), 8A Biomedical Grove, Immunos #05-13, Singapore, 138648 Singapore; 4 Infectious Diseases Translational Research Programme, Department of Microbiology and Immunology, Yong Loo Lin School of Medicine, National University of Singapore, Singapore, Singapore; 5 Lee Kong Chian School of Medicine, Nanyang Technological University, Singapore, Singapore; 6 Department of Pharmacy-Pharmaceutical Sciences, University of Bari Aldo Moro, via E. Orabona, 4, 70125 Bari, Italy; 7 Department of Biophysics and Physical Chemistry, Faculty of Pharmacy, Charles University, Heyrovského 1203, 500 03 Hradec Králové, Czech Republic

## Abstract

Understanding how antiviral compounds modulate infection dynamics is essential for the assessment and optimization of therapeutic candidates. Here, we combined mechanistic modeling with *in vitro* experimental data to quantify the antiviral efficacy of PB28, a high-affinity sigma receptor ligand, against SARS-CoV-2 infection in A549-ACE2 cells. Viral load measurements from both time-course and end-point assays across multiple PB28 concentrations were used to calibrate a viral dynamics model incorporating a Hill-type inhibitory function. This approach enabled the estimation of key viral life-cycle parameters – including infection rate, latent and infectious phase durations, and virus production rate – alongside parameters characterizing the action of PB28. Despite relying on minimal experimental input, characteristic of early-stage drug repurposing screens, the model provided accurate fits and robust parameter estimates. Our analysis shows that PB28 reduces viral production in a concentration-dependent manner with near-linear cooperativity and demonstrates how time-resolved modeling facilitates *post hoc* inference of an apparent, time-dependent $\text {IC}_{50}$. Notably, estimates of $\text {IC}_{50}$ from time-resolved infections were approximately twofold higher than those obtained from end-point assays. Overall, these results demonstrate that dynamic modeling facilitates more targeted characterization of pharmacodynamic parameters, including $\text {IC}_{50}$.

**Supplementary Information:**

The online version contains supplementary material available at 10.1007/s11538-026-01642-2.

## Introduction

The emergence of severe acute respiratory syndrome coronavirus 2 (SARS-CoV-2) in late 2019 and the subsequent global spread of coronavirus disease 2019 (COVID-19) triggered an unprecedented global effort to identify effective antiviral treatments. Given the urgency of the pandemic, much of the early therapeutic strategy focused on repurposing existing drugs, rather than developing new antiviral compounds (Gordon et al. 2020; Guy et al. 2020; Mslati et al. 2021; Bakowski et al. 2021; Han et al. 2021; Li et al. 2023; Liang et al. 2024; Singh et al. 2025). Repurposed drugs benefit from existing pharmacokinetic, safety, and manufacturing data, which can significantly accelerate the timeline for clinical use. A wide range of compounds have been evaluated through *in vitro* high-throughput screenings, structure-based docking studies, and hospital-based clinical trials to identify candidates capable of inhibiting SARS-CoV-2 infection and replication (Li et al. 2023; Consortium WST 2021; Chakraborty et al. 2021).

Antiviral strategies broadly fall into two main categories. First, direct-acting antivirals that target the virus itself, and second, host-directed therapies that modulate host cell processes essential for the viral life cycle (Kaufmann et al. 2018). Direct-acting antivirals typically inhibit key viral enzymes or structural proteins involved in viral replication cycle. While some drugs may target the viral RNA-dependent RNA polymerase, others may inhibit the viral main protease (Warren et al. 2016; Owen et al. 2021). Agents, which aim to directly suppress viral replication by disabling essential components of the viral machinery, are being actively developed as broad-spectrum therapeutics, targeting not only SARS-CoV-2 (Teoh et al. 2020; Schreiber and Ludwig 2025), but any member of coronavirus family (Laporte et al. 2025).

One notable host factor implicated in SARS-CoV-2 infection is the sigma-1 and sigma-2 receptors, which are primarily located at the endoplasmic reticulum and involved in calcium signaling, lipid metabolism, and cellular stress responses (Drewes et al. 2025; Izzo et al. 2020). Sigma receptor has been shown to interact with non-structural SARS-CoV-2 proteins, and modulation of its function can disrupt processes that support viral replication (Brimson et al. 2021; Abatematteo et al. 2023; Zhang et al. 2024). PB28, a subnanomolar ligand for both sigma-1 and sigma-2 receptors, emerged from high-throughput screening and computational analysis as a promising inhibitor of SARS-CoV-2 replication (Gordon et al. 2020; Abate et al. 2020; Song et al. 2025; Sauvat et al. 2020). Nevertheless, the *in vitro* antiviral effect of PB28 on the temporal progression of SARS-CoV-2 infection has not been properly studied and quantified. Traditionally used *in vitro* inhibition curves and single-point viral load assays may obscure the temporal dynamics of infection and drug action. Thus, a quantitative, mechanistic understanding of PB28 antiviral effects is essential for evaluating its potential for further preclinical development.

To describe the temporal effects of PB28 on SARS-CoV-2 infection *in vitro*, we performed a series of experimental infections of A549-ACE2 cells following treatments with different concentrations of PB28 and frequently measured viral levels over several days. Two concentrations of PB28, 0.5 $\upmu$M and 5 $\upmu$M, were used to treat A549-ACE2 cells to fully observe the inhibitory action of PB28 on the initial and late phase of SARS-CoV-2 infection. The efficacy of a drug to inhibit viral growth is usually determined from the concentration-response data in the form of the half-maximum inhibitory concentration, $\hbox {IC}_{50}$. Therefore, we measured the SARS-CoV-2 viral load in a series of end-point cell infections using a range of PB28 concentrations. Using data from both time-resolved and end-point experiments across a range of PB28 concentrations, we applied a mathematical model of *in vitro* viral infection incorporating target cell limitation (Baccam et al. 2006) and delayed virus production (Kakizoe et al. 2015; Bernhauerová et al. 2021). The model was calibrated within the Bayesian parameter estimation framework. Although SARS-CoV-2 *in vitro* infection and replication parameters have previously been determined in different cell lines, including A549-ACE2 cells (Bernhauerová et al. 2021; Staroverov et al. 2023, 2024), we additionally assessed the efficacy of PB28 in inhibiting virus production across a range of concentrations, determined the pharmacodynamic characteristics of PB28, including $\hbox {IC}_{50}$ and Hill coefficient values, and evaluated how PB28 alters SARS-CoV-2 infection dynamics in a time-dependent manner.

## Materials and Methods

### Cells

A549 cells stably expressing ACE2 (A549-ACE2, kindly provided by Dr. Olivier Schwartz), were propagated at $37^\circ$ C, 5% $\hbox {CO}_2$ in DMEM with L-glutamine (Gibco) supplemented with 10% FBS, penicillin-streptomycin and 20 $\upmu$g/mL blasticidin S.

### Virus

The SARS-CoV-2 strain used (BetaCoV/France/IDF0372/2020 strain) was propagated once in Vero-E6 cells and is a kind gift from the National Reference Centre for Respiratory Viruses at Institut Pasteur, Paris, originally supplied through the European Virus Archive goes Global platform.

### End-Point Experimental Infections

20,000 A549-ACE2 cells per well were seeded in a 96-well plate the day before infection and incubated at $37^\circ$ C, 5% $\hbox {CO}_2$. 2 h prior to infection, cells were treated either with vehicle DMSO, 0.01, 0.1, 0.2, 0.5, 2, or 10 $\upmu$M PB28. The amount of vehicle DMSO was kept constant in all conditions. At the time of infection, the medium was removed and replaced with 50 $\upmu$L virus-containing media (MOI 0.1 PFU per cell) and incubated at $37^\circ$ C for 1 h. Following this adsorption period, the virus-containing media was removed, cells washed three times with PBS and replaced with 100 $\upmu$L of either vehicle DMSO or PB28-containing media. At 73 h post-infection, 5 $\upmu$L of the supernatant from triplicate wells was removed for viral load analysis.

### Cell Viability Assays

Cell viability in drug-treated cells was measured using Alamar blue reagent (ThermoFisher). Briefly, 48 h post-treatment, the drug-containing medium was removed and replaced with Alamar blue and incubated for 1 h at $37^\circ$ C and fluorescence measured in a Tecan Infinity 2000 plate reader. Percentage viability was calculated relative to untreated cells (100% viability) and cells lysed with 20% ethanol (0% viability), included in each experiment.

### Time-Resolved Experimental Infections

400,000 A549-ACE2 cells per well were seeded in a 6-well plate the day before infection and incubated at $37^\circ$ C, 5% $\hbox {CO}_2$. 2h prior to infection, cells were treated either with vehicle DMSO, 0.5 $\upmu$M, 1 $\upmu$M, or 5 $\upmu$M PB28. At the time of infection, the medium was removed and replaced with 500 $\upmu$L virus-containing media (MOI 0.1 PFU per cell) and incubated at $37^\circ$ C for 1 h. Following this adsorption period, the virus-containing media was removed, cells washed three times with PBS and replaced with 2 mL of either vehicle DMSO, 0.5 $\upmu$M, 1 $\upmu$M, or 5 $\upmu$M PB28. At 17, 25, 49, 73, and 97 h post-infection, 50 $\upmu$L of the supernatant from triplicate wells was removed for viral load analysis. At each point, the medium removed was replaced with fresh drug- or vehicle-containing media.

### Virus Quantification

Viral load was assessed by RT-qPCR. Briefly, the cell culture supernatant was collected, heat inactivated at $95^\circ$ C for 5 minutes and used for RT-qPCR analysis. SARS-CoV-2 specific primers targeting the N gene region: 5’-TAATCAGACAAGGAACTGATTA-3’ (Forward) and 5’-CGAAGGTGTGACTTCCATG-3’ (Reverse) were used with the Luna Universal One-Step RT-qPCR Kit (New England Biolabs) in an Applied Biosystems QuantStudio 6 thermocycler, with the following cycling conditions: $55^\circ$ C for 10 min, $95^\circ$ C for 1 minute, and 40 cycles of $95^\circ$ C for 10 seconds, followed by $60^\circ$ C for 1 minute. The number of viral genomes is expressed as plaque forming unit equivalents per mL, $\hbox {PFU}_\text {e}$/mL, and was calculated by performing a standard curve with RNA derived from a viral stock with a known viral titer.

### Mathematical Model

#### Viral infection dynamics

We describe the *in vitro* infection dynamics of SARS-CoV-2 as in Bernhauerová et al. (2021) with the modification that all cell populations are expressed as proportions relative to the initial number of susceptible cells at time $t = 0$ h. Specifically, the proportion of suceptible cells, *S*, is infected with SARS-CoV-2 virus, *V* (measured in $\hbox {PFU}_\text {e}$/mL), at rate constant $\beta$ (measured in $\text {mL}/(\text {PFU}_\text {e}\times \text {h})$). We assume a delay between viral entry into the cell and the first release of newly produced viral particles (latent phase). This delay is modeled by separating the latent phase into $n_\text {L}$ stages. The proportion of latent cells in stage *i*, denoted $L_i,~i=1,\ldots ,n_\text {L}$, progresses through stages at rate constant $n_\text {L}/\tau _\text {L}$, where $\tau _\text {L}$ (measured in h) is the mean duration of the latent phase. A stage-structured latent phase was proposed in Kakizoe et al. (2015) and has been used to model *in vitro* virus spread (Beauchemin et al. 2019; Bernhauerová et al. 2020; Hurtado and Kirosingh 2019; Pinilla et al. 2012; Paradis et al. 2015). The proportion of cells in the final stage of the latent phase, $L_{n_\text {L}}$, becomes infectious at rate constant $1/\tau _I$, where $\tau _\text {I}$ (measured in h) is the mean duration of the infectious phase. The proportion of infectious cells, *I*, produces infectious virus at rate constant *p* (measured in $\text {PFU}_\text {e}/(\text {mL}\times \text {h})$). SARS-CoV-2 viral genomes remain stable for extended time periods (Bernhauerová et al. 2021; Utama et al. 2022). Since infectious viral load was approximated from RT-qPCR measurements of viral RNA, we do not assume degradation of viral particles. Following infection, cells are not assumed to proliferate. The full viral dynamics model is as follows:

1 $$\begin{aligned} \displaystyle {\frac{dS}{dt}}= &  - \beta \,V(t)\,S(t), \end{aligned}$$

2 $$\begin{aligned} \displaystyle {\frac{dL_1}{dt}}= &  \beta \,V(t)\,S(t) - \frac{n_\text {L}}{\tau _\text {L}}\,L_1(t) \end{aligned}$$

3 $$\begin{aligned} \displaystyle {\frac{dL_i}{dt}}= &  \frac{n_\text {L}}{\tau _\text {L}}\,\left( L_{i-1}(t) - L_i(t)\right) \, \text {for} ~i=2,\dots ,n_\text {L}, \end{aligned}$$

4 $$\begin{aligned} \displaystyle {\frac{dI}{dt}}= &  \frac{n_\text {L}}{\tau _\text {L}}\,L_{n_\text {L}}(t) - \frac{1}{\tau _\text {I}}\,I(t), \end{aligned}$$

5 $$\begin{aligned} \displaystyle {\frac{dV}{dt}}= &  (1-\epsilon )\,p\,I(t) - \omega (t)\,V(t). \end{aligned}$$

The initial conditions are set as follows: $S(0) = 1$, $L_{1, \ldots , n_L}(0) = 0$, $I(0) = 0$, $V(0) = V_\text {0;experiment}$, where ‘experiment’ stands for the infection carried out in 96-well (end-point infections) or 6-well (time-resolved infections) plates. The initial number of A549-ACE2 cells seeded was 20,000 cells per well in 96-well plates and 400,000 per well in 6-well plates; the cells doubled before infections were carried out. All infections were carried out at an $\text {MOI} = 0.1$ PFU per cell.

Following the experimental protocol, 0.05 mL of viral stock was used for end-point infections and 0.5 mL for time-resolved infections; the viral stock concentrations used for the respective infections were 80,000 PFU/mL and 160,000 PFU/mL. Since the concentration of cells at the time of infection was 40,000 cells/0.1 mL (equivalent to 400,000 cells/mL) per well in a 96-well plate, and 800,000 cells/2 mL (equivalent to 400,000 cells/mL) per well in a 6-well plate, we set $V_\text {0;96-well} = 8,000$ PFU/mL and $V_\text {0;6-well} = 16,000$ PFU/mL.

**Fig. 1:**
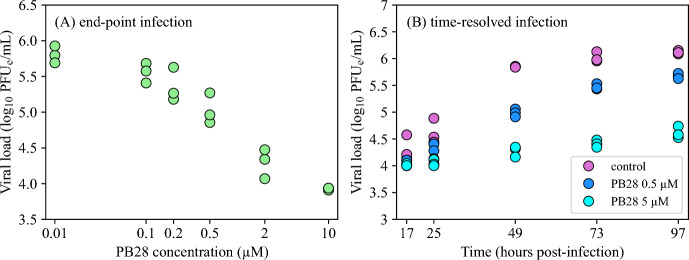
**Experimental SARS-CoV-2 infection of A549-ACE2 cells.** (A) Viral loads measured at 73 h post-infection at the indicated concentrations of PB28 in 96-well plates. (B) Viral loads measured at the indicated times post-infection in the control and following treatment with PB28 (purple) at concentrations of 0.5 (blue) and 5 $\upmu$M (cyan) in 6-well plates. Infections were performed at an MOI of 0.1 PFU/cell.

*In vitro* treatments with PB28 modulator have been shown to reduce viral load (Gordon et al. 2020; Abate et al. 2020). Following these results, we assume that PB28 reduces the rate at which virus is released by the proportion of infectious cells  *I*, by the factor $(1-\epsilon )$, where $\epsilon$ can vary between 0 (no inhibition of virus production) and 1 (complete inhibition of virus production). The efficacy of PB28 to inhibit virus production, $\epsilon$, is modelled by the Hill function

6 $$\begin{aligned} \epsilon = \frac{\epsilon _{\text {max}}\,C^{N_{\epsilon }}}{{IC}_{50,\epsilon }^{N_{\epsilon }} + C^{N_{\epsilon }}}, \end{aligned}$$

where $\epsilon _{\text {max}}$ is the maximum inhibition efficacy, *C* is PB28 concentration (measured in $\upmu$M), ${IC}_{50,\epsilon }$ is the concentration (measured in $\upmu$M) at which inhibition efficacy is $\epsilon _{\text {max}}/2$, and $N_{\epsilon }$ is the Hill coeficient. We assume that at high PB28 concentrations, the inhibitory effect on the virus is complete, thus $\epsilon _\text {max}=1$. This assumption was derived from the concentration-response curves obtained from experimental infections of PB28-treated A549-ACE2 cells with SARS-CoV-2 (Figure 1A). Specifically, PB28 at concentrations up to 10 $\upmu$M nearly completely suppressed viral accumulation at 73 h post-infection, yielding viral levels comparable to those observed at 17 h post-infection in the control as well as in cells pre-treated with 0.5 or 5 $\upmu$M PB28 (Figure 1B), when the virus had not yet begun to accumulate.

#### Washing rate, $\boldsymbol{\omega (t)}$

Viral load *V* in Equation (5) is reduced due to the one-time washing procedure 1 h after infection during which cells are washed off the excess virus that remained in the extracellular medium. We assume that the washing process occurs at the rate $\omega (t)$ and is described as

7 $$\begin{aligned} \omega (t) = \omega _0\frac{1}{\sqrt{2\pi \omega _d^2}}\exp \left( -\frac{(t - \omega _t)^2}{2\omega _d^2}\right) . \end{aligned}$$

This approach was proposed in Zitzmann et al. (2020) and applied in Bernhauerová et al. (2021) to capture the initial drop in the SARS-CoV-2 viral load. The parameter $\omega _0$ is the washing rate constant, $\omega _t$ is the timing of the washing process and was set to $\omega _t = 1$ h as in Bernhauerová et al. (2021). The parameter $\omega _d$ is the standard deviation of the washing process. Since cells were washed for approximately 3 minutes, $\omega _d$ was set to $\omega _d = 0.05$ h as in Bernhauerová et al. (2021).

### Parameter Estimation

Fitting was performed using the Python implementation of the Markov chain Monte Carlo method emcee that was proposed by Goodman and Weare (2010) and implemented in Foreman-Mackey et al. (2013). In what follows, all prior parameter distributions were assumed to be uniform.

#### SARS-CoV-2 infection dynamics

For estimation of the viral infection parameters $\beta$, $\tau _\text {L}$, $\tau _\text {I}$, *p*, and $n_\text {L}$ (Equations (1)–(5)), the parameter describing the washing process (Equation (7)), $\omega _0$, and the parameters characterizing the efficacy of PB28 to inhibit of virus production, $N_{\epsilon }$ and $IC_{50,\epsilon }$, we simultaneously fitted solutions of Equations (1)–(5) to the viral loads from end-point infections following treatments with PB28 at concentrations 0.01 $\upmu$M, 0.1 $\upmu$M, 0.2 $\upmu$M, 0.5 $\upmu$M, 2 $\upmu$M, and 10 $\upmu$M and to the viral loads from time-resolved infections either in the absence of PB28 or following treatments with PB28 at concentrations of 0.5 $\upmu$M and 5 $\upmu$M. To evaluate the goodness of the proposed fit of the predicted viral load, $\log _{10} V_{C_j}$, to the viral load data, $\log _{10}\text {data}_{i,C_j}^\text {virus}$, we maximized the logarithm of the Gaussian likelihood function

8 $$\begin{aligned} -\frac{1}{2}\left( L_\text {96-well} + L_\text {6-well}\right) , \end{aligned}$$

where

9 $$\begin{aligned} L_\text {96-well} = \sum _{i, j}\left[ \left( \frac{\log _{10}\text {data}^\text {virus}_{i,C_j} - \log _{10} V_{C_j}(\vec {q})}{\sigma ^\text {virus}}\right) ^2 + 2\,\ln \left( \sqrt{2\pi }\sigma ^\text {virus}\right) \right] , \end{aligned}$$

where $\vec {q}=\left( \beta , \tau _\text {L}, \tau _\text {I}, p, n_\text {L}, N_{\epsilon }, IC_{50,\epsilon }\right)$, taking into account viral load data obtained from three experimental replicates, $i\in \{1,2,3\}$, and from six infection experiments performed in 96-well plates following treatment with PB28 at concentrations $C_j$, $j\in \{1,\ldots ,6\}$. The parameter $\sigma ^\text {virus}$ in Equation (9) denotes the mean of the standard deviations of the viral infection data $\log _{10}\text {data}^\text {virus}_{C_j}$ computed across all PB28 concentrations $C_j$. Moreover,

10 $$\begin{aligned} L_\text {6-well} = \sum _{i, j, k}\left[ \left( \frac{\log _{10}\text {data}^\text {virus}_{i,C_j}(t_k) - \log _{10} V_{C_j}(t_{k}, \vec {q})}{\sigma ^\text {virus}_{C_j}}\right) ^2 + 2\,\ln \left( \sqrt{2\pi }\sigma ^\text {virus}_{C_j}\right) \right] , \end{aligned}$$

where infections were performed in three experimental replicates, $i\in \{1,2,3\}$, in 6-well plates, either in the absence of PB28, $C_1=0$ $\upmu$M, or following treatment with PB28 at concentrations $C_j$, $j\in \{2,3\}$. Viral load was measured at five time points, $t_k\in \{17~\text {h}, 25~\text {h}, 49~\text {h}, 73~\text {h}, 97~\text {h}\}$, with $k\in \{1,\ldots ,5\}$. The parameter $\sigma ^\text {virus}_{C_j}$ denotes the mean of the standard deviations of the viral infection data $\log _{10}\text {data}^\text {virus}_{C_j}(t_k)$ computed across all measured time points for each PB28 concentration $C_j$. All else remains as in Equation (9).

#### SARS-CoV-2 inhibition

To estimate PB28 parameters characterizing the concentration-dependent decrease in viral yield (Figure 1A), we fitted the equation

11 $$\begin{aligned} \log _{10}V = \log _{10}\left( V_\text {min} + \frac{V_\text {max} - V_\text {min}}{1 + 10^{N\,(\log _{10}C - \log _{10}IC_{50})}}\right) , \end{aligned}$$

to the viral loads from end-point infections following treatment with PB28 at the indicated concentrations. In Equation (11), $V_\text {min}$ and $V_\text {max}$ are plateau viral levels at the minimum and maximum PB28 concentrations, *C*, respectively, ${IC}_{50}$ is the concentration (measured in $\upmu$M) at which inhibition efficacy is 1/2, and *N* is the Hill coefficient. To evaluate the goodness of the proposed fit of the predicted viral levels, $\log _{10}V$, to the viral load data, $\log _{10}\text {data}^\text {virus}_{i}$, we maximized the logarithm of the Gaussian likelihood function

12 $$\begin{aligned} -\frac{1}{2}\sum _{i, j}\left[ \left( \frac{\log _{10}\text {data}^\text {virus}_{i}(C_j) - \log _{10} V(C_j, \vec {q})}{\sigma ^\text {virus}_{C_j}}\right) ^2 + 2\,\ln \left( \sqrt{2\pi }\sigma ^\text {virus}_{C_j}\right) \right] , \end{aligned}$$

where $\vec {q}=\left( V_\text {min},V_\text {max},IC_{50},N\right)$, taking into account viral load data obtained from three experimental replicates, $i\in \{1,2,3\}$, following treatment with PB28 at concentrations $C_j$, $j\in \{1,\ldots ,6\}$. The parameter $\sigma ^\text {virus}_{C_j}$ denotes the standard deviation of the viral load data $\log _{10}\text {data}^\text {virus}(C_j)$.

#### A549-ACE2 viability

**Fig. 2:**
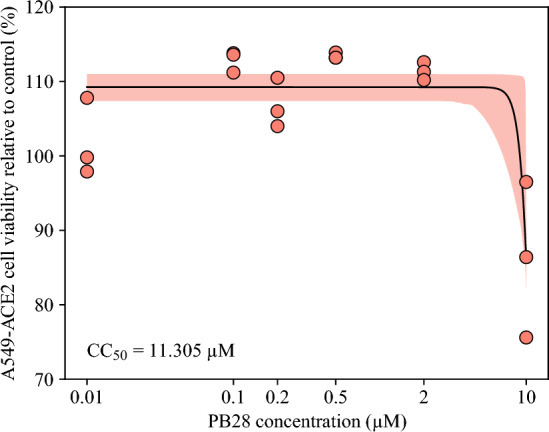
**A549-ACE2 cell viability as a function of PB28 concentration.** Equation (13) was fitted to the relative percentages of viable cells (relative to the control) using MCMC (details are in Materials and Methods). A total of 6 independent chains were run for 20,000 steps, with a burn-in of 10,000 steps. Thinning was applied by accepting every 10th step. A total of 6,000 accepted parameter sets were considered. The maximum likelihood (best-fit) solution is displayed as a black solid line. The 95% credible bands are displayed as filled areas. Parameter values are in Table 1.

To estimate parameters describing the concentration-dependent viability of A549-ACE2 cells, we fitted the equation

13 $$\begin{aligned} A = A_\text {min} + \frac{A_\text {max} - A_\text {min}}{1 + 10^{N_\text {A}\,(\log _{10}C - \log _{10}CC_{50})}}, \end{aligned}$$

to the relative cell viability at 72 h following treatment with PB28, normalized to the control, at the indicated concentrations (Figure 2). In Equation (13), $A_\text {min}$ and $A_\text {max}$ are plateau relative cell viabilty levels at the minimum and maximum PB28 concentrations, *C*, respectively, and $CC_{50}$ is the PB28 half-maximum cytotoxic/cytostatic constant and $N_\text {A}$ is the Hill coeficient. We assume that for high concentrations of PB28, $C\rightarrow \infty$, viability goes to zero and thus $A_\text {min} = 0$.

To evaluate the goodness of the proposed fit of the predicted cell viability levels, *A*, to the cell viability data, $\text {data}_{i}^\text {A}$, we maximized the logarithm of the Gaussian likelihood function

14 $$\begin{aligned} -\frac{1}{2}\sum _{i, j}\left[ \left( \frac{\text {data}_{i}^\text {A}(C_j) - A(C_j, \vec {q})}{\sigma ^\text {A}}\right) ^2 + 2\,\ln \left( \sqrt{2\pi }\sigma ^\text {A}\right) \right] , \end{aligned}$$

where $\vec {q}=\left( A_\text {max},CC_{50},N_\text {A}\right)$, taking into account cell viability data obtained from three experimental replicates, $i\in \{1,2,3\}$, following treatment with PB28 at concentrations $C_j$, $j\in \{1,\ldots ,6\}$. The parameter $\sigma ^\text {A}$ denotes the mean of the standard deviations of the cell viability $\text {data}^\text {A}(C_j)$ computed across all PB28 concentrations $C_j$.

### Statistical Analysis

The Mann-Whitney U test was used to determine statistically significant differences between the estimated parameters using the mannwhitneyu function from the scipy package in Python, with the significance level set at p-value<0.05.

**Table 1:** PB28 parameter estimates obtained from A549-ACE2 cell viability assay (Equation (13)).

Parameter	Description	Units	ML value	95% CI
$\hbox {A}_\text {min}$	Plateu at minimum	%	0	fixed
$\hbox {A}_\text {max}$	Plateu at maximum	%	109.25	[107.40, 110.99]
$CC_{50}$	PB28 half-maximum cytotoxic/cytostatic constant	$\upmu$M	11.305	[10.030, 16.023]
$N_\text {A}$	Hill coeficient	-	10.73	[2.83, 438.71]

## Results

### SARS-CoV-2 Experimental Infections in PB28-Treated A549-ACE2 Cells

A549-ACE2 cells were infected with SARS-CoV-2 at an MOI of 0.1 PFU per cell. We performed two types of infections: (a) time-resolved infections in 6-well plates, in which quantifications of viral load were performed at 17, 25, 49, 73, and 97 h post-infection; and (b) end-point infections in 96-well plates, in which quantifications of viral load were performed at time 73 h post infection (details are in Materials and Methods). The 96-well format was specifically used to examine the concentration-response relationship between viral load and PB28 concentration, whereas the 6-well format enabled detailed temporal analysis of infection dynamics. These complementary experimental setups enabled both a detailed reconstruction of infection kinetics and a quantitative assessment of PB28 action through mathematical modeling.

A549-ACE2 cells were cultured either in the absence or presence of PB28 at various concentrations. In 96-well plates, cells were treated with PB28 at 0.01, 0.1, 0.2, 0.5, 2, and 10 $\upmu$M (Figure 1A). In 6-well plates, cells were left untreated (hereafter referred to as the control) or treated with PB28 at 0.5 and 5 $\upmu$M (Figure 1B). In the control condition, viral load began to plateau at 97 h post-infection. PB28 treatment resulted in a concentration-dependent reduction in viral load, consistent with previous reports (Gordon et al. 2020; Abate et al. 2020; Song et al. 2025).

We assessed the impact of PB28 on A549-ACE2 cell viability following treatment with the indicated concentrations of PB28 (Figure 2). Using a Markov chain Monte Carlo (MCMC) approach, we fitted Equation (13) to the viability data and estimated the half cytotoxic/cytostatic concentration of PB28, $CC_{50}$, to be 11.305 $\upmu$M (95% credible interval (CI): [10.030; 16.023] $\upmu$M), which was substantially lower than the range of 38.79–63.35 $\upmu$M determined for Vero-E6, ST and HCT-8 cells in Song et al. (2025). We further estimated the Hill coefficient $N_\text {A}$ to be 10.73, with a wide 95% CI, indicating that the viability data were insufficient to reliably quantify this parameter (Table 2). The MCMC posterior distributions and the correlation plots for all viability parameters are shown in Figure S1 in Online Resource 1. Because the A549-ACE2 cell population remained at or above control levels across all tested PB28 concentrations (Figure 2), we assumed in subsequent analyses that PB28 did not affect cell viability.

### Estimation of SARS-CoV-2 Infection Parameters

To recover the SARS-CoV-2 infection parameters, we simultaneously fitted the viral dynamics model (Equations (1)–(5)) to viral load data from both end-point and time-resolved infections using MCMC. The parameters estimated included the infection rate constant, $\beta$; the number of compartments of the latent phase, $n_\text {L}$; the duration of the latent phase, $\tau _L$; the duration of the infectious phase, $\tau _I$; and the virus production rate constant, *p*. In addition, we estimated the parameter constant characterizing the washing process, $\omega _0$. The summary of the parameter values and their 95% CIs is given in Table 2. The posterior parameter distributions obtained from the MCMC analysis, along with the parameter correlation plots, are shown in Figure S2 in Online Resource 1.

**Fig. 3:**
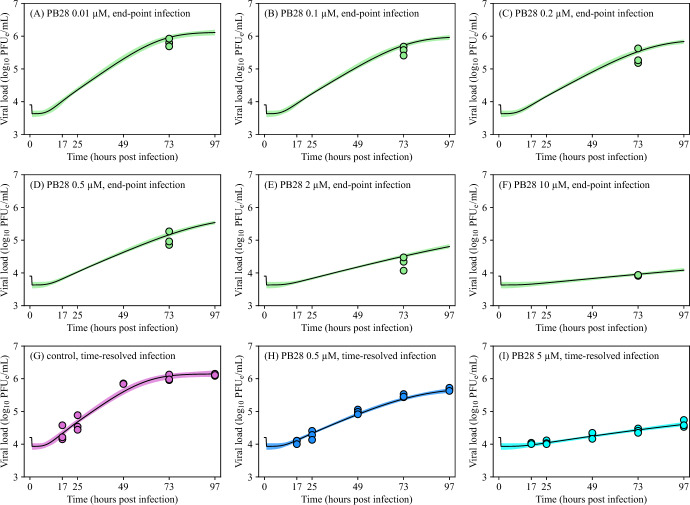
**Fits of the viral dynamics model to SARS-CoV-2 viral load data.** Equations (1)–(5) were simultaneously fitted to SARS-CoV-2 viral loads obtained from end-point (panels (A)–(F)) and time-resolved (panels (G)–(I)) experimental infections using MCMC (details are in Materials and Methods). A total of 16 independent chains were run for 20,000 steps, with a burn-in of 10,000 steps. Thinning was applied by accepting every 10th step. A total of 16,000 accepted parameter sets were considered. The maximum likelihood (best-fit) solutions are displayed as black solid lines. The 95% credible bands are displayed as filled areas. Legend as in Figure 1.

**Fig. 4:**
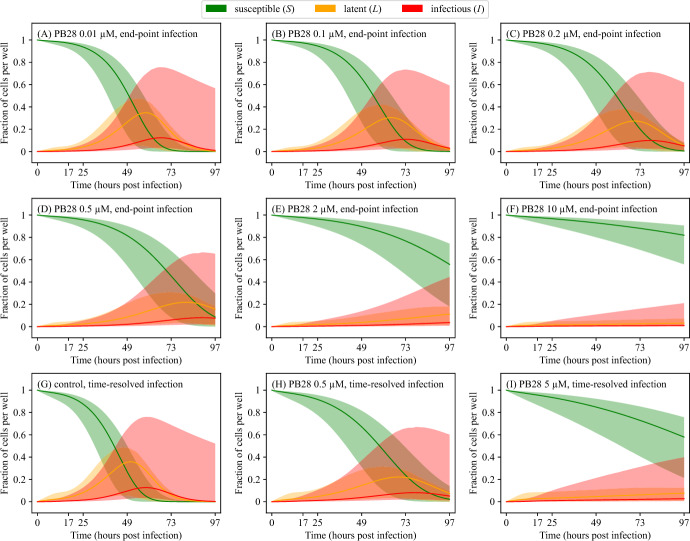
**A549-ACE2 cell dynamics.** Proportions of cells associated with fits of Equations (1)–(5) to SARS-CoV-2 viral loads obtained from end-point and time-resolved experimental infections. Proportions of cells were calculated using the MCMC-accepted parameters, as described in Figure 3. The maximum likelihood (best-fit) solutions are displayed as solid lines. The 95% credible bands are displayed as filled areas.

The fits of the solutions of Equations (1)–(5) to viral load data from the end-point and time-resolved infections are in Figures 3A–F and 3H–I, respectively. These predictions were in good agreement with the experimental data, suggesting that the estimated viral parameters are robust across different experimental setups. Figure 4 shows the corresponding dynamics of the proportions of susceptible, latent, and infectious A549-ACE2 cells. As the concentration of PB28 increased, less virus was released by infectious cells, resulting in a higher overall proportion of uninfected cells.

We estimated that SARS-CoV-2 infected susceptible cells at the rate constant, $\beta$, of $2.85\times 10^{-7}$ mL/($\hbox {PFU}_\text {e}\times$ h) (95% CI: [1.49; 7.96] $\times 10^{-7}$ mL/($\hbox {PFU}_\text {e}\times$ h)). Previously reported estimates of $\beta$ varied significantly. In Caco-2, Calu-3, and A549-ACE2 cells, $\beta$ was estimated to be one to two orders of magnitude lower (Bernhauerová et al. 2021; Staroverov et al. 2023, 2024), whereas in Vero-E6 cells it was approximately one order of magnitude higher (Bernhauerová et al. 2021). These differences may be attributed to different viral strains (wild type, Delta, and Omicron in Staroverov et al. (2023, 2024)) versus the Beta strain used in this study and in Bernhauerová et al. (2021), as well as to differences in the cell lines used to propagate SARS-CoV-2.

The duration of the latent phase, $\tau _\text {L}$, was estimated to be 11.96 h (95% CI: [1.94; 16.77] h), which is comparable to the value of 7.36 h reported for infections of Caco-2 and Calu-3 cells in Staroverov et al. (2023, 2024). However, it was lower—and with non-overlapping 95% CIs—than the estimates of 42.74 h and 27.07 h reported for infections of A549-ACE2 and Vero-E6 cells, respectively. The number of compartments characterizing the latent phase, $n_\text {L}$, was allowed vary from 1 to 50. However, the posterior distribution of $n_\text {L}$ yielded by MCMC analysis remained uniform, as the data did not provide enough information to update the prior.

As in our previous study Bernhauerová et al. (2021), we imposed an upper bound of 100 h on the duration of the infectious phase, $\tau _\text {I}$, to prevent MCMC chains from converging to ever-increasing—and thus unrealistic—values of $\tau _\text {I}$. Combined with a strong positive correlation between the virus production rate constant, *p*, and $\tau _\text {I}$, reported in previous studies (Beauchemin et al. 2019; Bernhauerová et al. 2020), neither parameter could be reliably estimated. Consequently, the 95% CIs around the best-fit solutions for the proportions of susceptible, latent, and infected cells were relatively wide (Figure 4).

The parameter controlling the strength of the washing process, $\omega _0$, was estimated to be 0.62 (95% CI: [0.41; 0.86]), which is lower—and had non-overlapping 95% CIs—compared with the values of 2.0 and 2.18 reported in Bernhauerová et al. (2021).

To further examine the influence of parameters on our results, we performed a local sensitivity analysis, in which input parameters were perturbed one at a time by $\pm ~50\%$ from their best-fit estimates, to evaluate how each affects the model output. Indeed, SARS-CoV-2 infection dynamics was sensitive to perturbations in both, $\tau _\text {I}$ and *p* (Figures S3 and S4, respectively, in Online Resource 1). Moreover, SARS-CoV-2 viral dynamics was also sensitive to changes in $\beta$ and $\tau _\text {L}$ (Figures S5 and S6, respectively, in Online Resource 1), mildly sensitive to changes in $\omega _0$ and insensitive to changes in $n_\text {L}$ (Figures S7 and S8, respectively, in Online Resource 1).

**Fig. 5:**
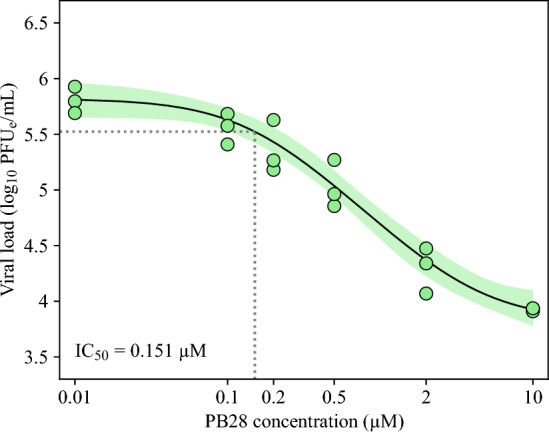
$\hbox {IC}_{50}$ **predicted from end-point infection assay. ** Equation (11) was fitted to viral loads obtained from end-point experimental infections using MCMC (details are in Materials and Methods). A total of 8 independent chains were run for 20,000 steps, with a burn-in of 10,000 steps. Thinning was applied by accepting every 5th step. A total of 16,000 accepted parameter sets were considered. The maximum likelihood (best-fit) solution is displayed as a black solid line. The 95% credible bands are displayed as filled areas. Legend as in Figure 1.

**Fig. 6:**
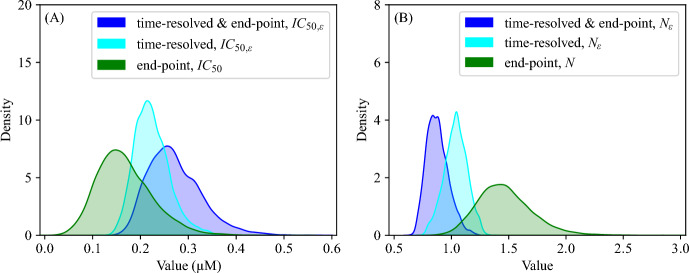
**PB28 parameters for time-resolved and end-point experimental infections.** Parameter posterior histograms of (A) PB28 half-maximum inhibition constant and (B) Hill coefficient estimated from fitting the viral dynamics model (Equations (1)–(5)) to SARS-CoV-2 viral loads obtained from end-point and time-resolved experimental infections (blue), time-resolved experimental infections (cyan), and from fitting Equation (11) to SARS-CoV-2 viral loads obtained from end-point experimental infections (green). Fitting was performed using MCMC, as described in Figures 3 and 5.

### Estimation of PB28 Parameters

We estimated two parameters characterizing the degree of inhibition of SARS-CoV-2 by PB28, $\epsilon$ (Equation (6)): the half maximal inhibitory concentration, $IC_{50,\epsilon }$, and the Hill coefficient, which accounts for cooperativity in PB28 effect, $N_\epsilon$. In the time-resolved experimental infection scheme, we estimated $IC_{50,\epsilon }$ to be 0.257 $\upmu$M (95% CI: [0.186; 0.401] $\upmu$M) and $N_\epsilon$ to be 0.86 (95% CI: [0.72; 1.01]), favoring mildly negative cooperativity in PB28 effect on SARS-CoV-2 infection dynamics. In the end-point experimental infection scheme, we fitted Equation (11) to the PB28 concentration-response curve of viral load (Figure 5, details are in Materials and Methods) and estimated $IC_{50}$ to be 0.151 $\upmu$M (95% CI: [0.073; 0.299] $\upmu$M), with an associated best-fit $IC_{90}$ of 0.773 $\upmu$M. This value is higher than the reported $IC_{90}$ of 0.278 $\upmu$M in Vero-E6 (Gordon et al. 2020) and lower than the reported $IC_{50}$ values of 1.4–4.12 $\upmu$M in Vero-E6, ST, and HCT-8 cells (Song et al. 2025). Thus, the efficacy of PB28 in inhibiting SARS-CoV-2 accumulation may vary significantly across cell lines. The Hill coefficient, *N*, was estimated to be 1.41 (95% CI: [1.06; 2.01]), suggesting mildly positive cooperativity in the PB28 effect on SARS-CoV-2 infection dynamics.

The differences between $IC_{50,\epsilon }$ and $IC_{50}$, as well as between $N_\epsilon$ and *N*, for the time-resolved and end-point experimental infection schemes, respectively, were statistically significant (p-value $< 10^{-5}$). The corresponding values are shown as posterior parameter distributions in Figure 6 and listed in Tables 1 and 3. The parameter correlation plots for both the end-point and time-resolved experimental infection schemes are displayed in Figures S2 and S11 in Online Resource 1.

**Table 2:** SARS-CoV-2 and PB28 parameter estimates obtained from infection of A549-ACE2 cells (Equations (1)–(5)).

Parameter	Description	Units	ML value		95% CI	
			End-point & time-resolved	Time-resolved	End-point & time-resolved	Time-resolved
$\beta$	infection rate constant	mL/($\hbox {PFU}_\text {e}\times$h)	2.85$\times 10^{-7}$	7.28$\times 10^{-7}$	$[1.49, 7.96]\times 10^{-7}$	$[2.97, 22.86]\times 10^{-7}$
$\tau _\text {L}$	duration of latent phase	h	11.96	22.60	[1.94, 16.77]	[7.16, 44.06]
$\tau _\text {I}$	duration of infectious phase	h	4.50	2.93	[1.12, 85.51]	[1.30, 83.38]
$n_\text {L}$	number of latent phase compartments	-	4	5	[2, 48]	[3, 48]
*p*	virus production rate constant	$\hbox {PFU}_{\text {e}}$/(h$\times$mL)	3.09$\times 10^{5}$	5.15$\times 10^{5}$	$[0.38, 11.77]\times 10^{5}$	$[0.39, 12.60]\times 10^{5}$
$\omega _0$	washing rate constant	-	0.62	0.54	[0.41, 0.86]	[0.37, 0.76]
$\epsilon _{\text {max}}$	maximum inhibition efficacy	-	1	1	fixed	fixed
$IC_{50,\epsilon }$	PB28 half-maximum inhibition constant	$\upmu$M	0.257	0.21	[0.186, 0.401]	[0.16, 0.31]
$N_{\epsilon }$	Hill coeficient	-	0.86	1.03	[0.72, 1.09]	[0.83, 1.23]

**Table 3:** PB28 parameter estimates obtained from SARS-CoV-2 inhibition assay (Equation (11)).

Parameter	Description	Units	ML value	95% CI
$V_\text {min}$	Plateu at minimum	$\hbox {PFU}_{\text {e}}$/mL	6.68$\times 10^{3}$	[2.13, 11.61]$\times 10^{3}$
$V_\text {max}$	Plateu at maximum	$\hbox {PFU}_{\text {e}}$/mL	6.61$\times 10^{5}$	[4.51, 9.88]$\times 10^{5}$
$IC_{50}$	PB28 half-inhibition constant	$\upmu$M	0.151	[0.073, 0.299]
*N*	Hill coeficient	-	1.41	[1.06, 2.01]

### Validation of Predictions from Time-Resolved Infections Against end-Point Infections

To evaluate the robustness of the parameter estimates, we fitted Equations (1)–(5) exclusively to viral load data from time-resolved infections (Figure S12G–I in Online Resource 1) and used the resulting parameters to simulate SARS-CoV-2 infection dynamics in PB28-treated A549-ACE2 cells in 96-well plates (Figure S12A–F in Online Resource 1) to compare the model-predicted viral loads for end-point infections with experimentally measured end-point viral loads. Although these predicted viral loads were slightly higher than those obtained from simultaneous fitting of time-resolved and end-point datasets (Figure 3), they remained in good agreement with the experimental observations. These slight discrepancies arose from differences in the parameter estimates obtained under the two fitting configurations (Table 2). In particular, the higher estimated infection rate constant, $\beta = 7.28 \times 10^{-7}$ mL/($\hbox {PFU}_\text {e}\times$h), led to a more rapid transition of susceptible cells into the latent stage (Figure S13 in Online Resource 1). The duration of latent phase, $\tau _\text {L}$, was estimated to be 22.60 h, nearly twice the value obtained from simultaneous fitting of time-resolved and end-point datasets; however, this difference was not visually discernible from the fits to time-resolved viral load data alone. Although all model parameter estimates differed significantly between fitting configurations (p-value $<10^{-5}$), the estimates for the number of latent-phase compartments, $n_\text {L}$, the virus production rate constant, *p*, and the washing rate constant, $\omega _0$, showed substantial overlap in their 95% CIs (Table 2).

The estimated parameters describing PB28-mediated inhibition of SARS-CoV-2—the half-maximal inhibitory concentration, $IC_{50,\epsilon }$, and the Hill coefficient, $N_\epsilon$—were intermediate between the values obtained from simultaneous fitting of time-resolved and end-point datasets and those derived from fitting the concentration–response curve (Equation (11)) to end-point infections (Figure 6). The best-fit estimate of $IC_{50,\epsilon }$ was 0.21 $\upmu$M, which was slightly lower than the value obtained from simultaneous fitting of time-resolved and end-point datasets (0.257 $\upmu$M), with broadly overlapping 95% CIs. The best-fit estimate of $N_\epsilon$ was 1.03, which was slightly higher than the value obtained from simultaneous fitting of time-resolved and end-point datasets (0.86), also with broadly overlapping 95% CIs. These results indicate that the viral dynamics model, when applied to time-resolved datasets alone, may be sufficient to reliably estimate both $IC_{50,\epsilon }$ and $N_\epsilon$.

### Time-Dependent Prediction of PB28 $\text {IC}_{50}$

**Fig. 7:**
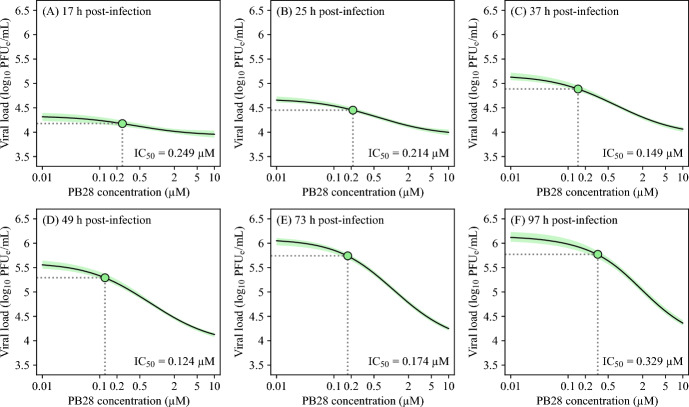
**Time-dependent changes in PB28** $\hbox {IC}_{50}$ **during SARS-CoV-2 infection of A549-ACE2 cells.** At 17, 25, 37, 49, 73, and 97 h post-treatment with PB28, the solution of the viral dynamics model (Equations (1)–(5)) was evaluated across a range of PB28 concentrations using parameter values in Table 2 to generate synthetic concentration–response curves. The maximum likelihood (best-fit) solution is displayed as a black solid line. The 95% credible bands are displayed as filled areas.

Since $\text {IC}_{50}$ values are predicted from concentration-response curves of end-point infections, typically at 48 or 72 h post-infection, they can vary due to the nonlinear progression of infection or delayed drug effects. To investigate whether $IC_{50}$ depends on the timing of the infection end-point, we evaluated viral load levels for each MCMC-accepted parameter set across the indicated range of PB28 concentrations (Figure 7). These evaluations were performed for end-points 17, 25, 37, 49, 73, and 97 h post-infection.

The values of $IC_{50}$ at each indicated end-point were obtained by fitting Equation (11) to the model-predicted viral loads associated with the best-fit parameters (Table 2) using the Python’s least_squares function for least-squares minimization. All parameters in Equation (11) were estimated. Our simulations revealed that the predicted PB28 $IC_{50}$ varied with the end-point time, exhibiting weaker inhibitory effects at early and late times and maximal inhibitory effect at intermediate times. Specifically, $IC_{50}$ decreased from 0.249 $\upmu$M at 17 h post-infection to 0.124 $\upmu$M at 49 h post-infection, increasing again to 0.329 $\upmu$M at 97 h post-infection.

## Discussion

Despite extensive global deployment of COVID-19 vaccines, the continual emergence of immune-escape SARS-CoV-2 variants and the long-term negative health effects associated with post-acute infection present significant challenges to public health (Markov et al. 2023). These factors highlight the critical need for the development of broad-spectrum antiviral therapeutics, particularly those leveraging host-targeted strategies, to mitigate current and future coronavirus outbreaks. High-throughput drug repurposing screens have identified potential therapeutic candidates against SARS-CoV-2 infection (Gordon et al. 2020). Most efforts have centered on direct-acting antivirals, particularly nucleoside analogs targeting the viral RNA-dependent RNA polymerase, such as remdesivir. However, recent evidence suggests that these agents may promote intra-host genomic diversity, potentially accelerating the emergence of novel variants with fixed mutations (Heyer et al. 2022). Increasing attention has therefore turned to therapeutics that modulate host cellular pathways critical to SARS-CoV-2 pathogenesis. The sigma-1 receptor has been implicated in viral entry and replication, and PB28, a selective sigma-1 receptor antagonist, has demonstrated potent antiviral activity *in vitro* (Gordon et al. 2020). Nevertheless, the molecular mechanisms underlying PB28’s efficacy, particularly against emerging SARS-CoV-2 variants, remain poorly understood.

In this study, we applied a mechanistic model of viral infection dynamics to describe SARS-CoV-2 infection of A549-ACE2 cells, explicitly incorporating the antiviral activity of PB28, and fitted the model to experimental viral load data. Two infection scenarios were examined: (a) time-resolved infections to characterize the temporal effects of PB28 on SARS-CoV-2 replication, and (b) end-point infections, commonly used to determine drug-related parameters from concentration–response curves. We found that the estimated $\text {IC}_{50}$ value varied depending on the infection scenario, with $IC_{50}$ derived from the end-point assay being approximately half that obtained from the time-resolved infection, $IC_{50,\epsilon }$. Although this difference was statistically significant, the 95% credible intervals for $IC_{50,\epsilon }$ and $IC_{50}$ notably overlapped. This discrepancy likely arises from the greater complexity of the time-resolved infection, which accounts for the dynamic progression of SARS-CoV-2 infection throughout all measured time points rather than a single end-point. To support this conclusion, we assessed the robustness of parameter inference from time-resolved infection data by excluding the end-point datasets and refitting the viral dynamics model using only the time-resolved measurements. The resulting estimates of $IC_{50,\epsilon }$ exhibited substantial overlap in their 95% credible intervals, indicating that the inference of this parameter is stable with respect to the exclusion of end-point infection data. Although different analytical frameworks yielded different estimates, they are best viewed as complementary: endpoint analyses yield comparative snapshots of antiviral activity, whereas dynamic models reveal the underlying time structure and may offer a framework for formulating and evaluating mechanistic hypotheses.

Estimates of $\text {IC}_{50}$ may also vary in response to differences in viral kinetics among biological replicates, arising from variability in underlying SARS-CoV-2 dynamic parameters. Indeed, key parameters such as the infection rate constant, $\beta$, and the latent-phase duration, $\tau _\text {L}$, have been shown to differ across cell lines and viral strains (Bernhauerová et al. 2021; Staroverov et al. 2023, 2024). We showed that these parameters vary also among replicates using the same cell line and viral strain (Bernhauerová et al. 2021). We argue that such differences should not be interpreted as mutually inconsistent results, but rather as context-dependent realizations of a common underlying dynamical system. For *in vivo* translation, isolated $\text {IC}_{50}$ values obtained from heterogeneous *in vitro* settings should not be directly compared or incorporated into pharmacodynamic models without accounting for their experimental context. Instead, characterizing how $\text {IC}_{50}$ depends on experimental and biological conditions is more informative than relying on a single point estimate, and meaningful translation to *in vivo* systems requires integrating these systematic patterns rather than using absolute values alone.

Using the estimates from the viral infection model, we predicted $IC_{50}$ at different end-points in time. Figure 7 visualizes how $IC_{50}$ decreases over time, from approximately 0.249 $\upmu$M at 17 h to 0.124 $\upmu$M at 49 h post-infection, before rising again to 0.329 $\upmu$M at 97 h post-infection. The lower $IC_{50}$ values observed at early end-points in time may reflect the lag between viral inoculation at $t=0$ h and the initial release of virions, during which not all cells are infected or actively producing virus (Figure 4). The transient increase in inhibitory potency may reflect the progressive synchronization of viral replication across the cell population, potentially coupled with PB28’s modulation of post-entry host pathways, such as trafficking or autophagy. The reduction in inhibitory effect observed at later time points is likely driven by the dynamic interaction between infection progression and drug action-such as drug effect saturation, loss of virus-producing cells, or modeling assumptions like constant viral stability-rather than by any change in the biochemical affinity of PB28. These hypotheses regarding transient changes in $IC_{50}$ values could be experimentally tested by measuring the PB28 concentration-dependent response in viral load at multiple time points, e.g., 25 h, 49 h, and 97 h, and across different multiplicities of infection, both lower and higher, to confirm that these transient changes reflect distinct stages of the viral replication cycle. More generally, it is desirable to validate the dependence of $\text {IC}_{50}$ on the time of measurement for different viruses and drugs with distinct mechanisms of action (e.g., entry inhibitors, polymerase inhibitors, protease inhibitors), as these factors may influence delays in viral production and the timing of the peak of viral infection, between which the rate of change in viral load is maximal (i.e., the derivative is highest). Our findings thus underscore the value of incorporating temporal resolution of an *in vitro* viral infection into pharmacodynamic assessments.

Time dependence of $\text {IC}_{50}$ values has also been reported in chemotherapy assays (Murphy et al. 2020; Sánchez-Díez et al. 2025) and in cytochrome P450 (CYP) enzyme inhibition assays (Krippendorff et al. 2009). Murphy et al. (2020) systematically investigated how the timing of measurements influences estimates of $\text {IC}_{50}$ and $\epsilon _{\text {max}}$ (the maximal drug effect) in chemotherapy assays using several ODE-based tumor growth models. Their results showed that $\text {IC}_{50}$ can shift substantially over time—sometimes by orders of magnitude—solely due to the dynamical properties of the underlying biological system and the choice of sampling time point. Likewise, Krippendorff et al. (2009) demonstrated that $\text {IC}_{50}$ values obtained from a mathematical model of catalytic enzyme reactions with mechanism-based inhibition are inherently time-dependent. These predictions were further supported experimentally using fluorimetric assay data from well-characterized mechanism-based inhibitors of CYP450 isoforms 1A2 and 3A4, where a systematic shift toward lower $\text {IC}_{50}$ values was observed for catalytic activity assays. These examples illustrate that $\text {IC}_{50}$ is not an intrinsic property of a drug alone, but rather an emergent quantity shaped by system dynamics and the timing of measurement. Our findings thus reinforce this concept in the context of viral replication and emphasize that assessing antiviral activity at a single fixed time point can bias potency estimates and complicate comparisons across experiments, cell lines, or compounds.

PB28 has been reported to enhance cell viability during SARS-CoV-2 infection in a concentration-dependent manner (Song et al. 2025). However, our modeling results indicate that this apparent cytoprotective effect is more likely attributable to reduced viral burden at higher PB28 concentrations, thereby limiting the infection of susceptible cells (Figure 4). The mathematical model is based on the assumption that PB28 primarily acts by reducing viral production, which may represent an oversimplification of its potentially multifaceted mechanisms. The exact mechanism by which PB28 suppresses SARS-CoV-2 replication remains unresolved. It has been suggested that its antiviral activity is primarily mediated through off-target, host-directed mechanisms rather than through its canonical pharmacological target, the sigma-1 receptor. PB28 and similar compounds are thought to interfere with viral replication by perturbing acidophilic host organelles, including autophagosomes, endosomes, and lysosomes (Sauvat et al. 2020). However, Song et al. (2025) reported that SARS-CoV-2 replication was already substantially reduced in sigma-1 receptor knockout Vero-E6 cells, and PB28 treatment did not produce additional inhibitory effects beyond the knockout alone. These findings indicate that PB28’s antiviral effect is likely mediated by modulation of sigma-1 receptor–dependent host processes rather than entirely unrelated off-target effects.

Nevertheless, off-target *in vitro* antiviral effects of sigma-1 receptor ligands—such as drug-induced phospholipidosis, modulation of ER stress, altered autophagy, or disruption of host pathways that limit viral replication—cannot be excluded (Ostrov et al. 2021; Brimson et al. 2021). Several sigma-1 receptor ligands have been shown to reduce SARS-CoV-2–induced cytopathic effects in Vero-E6 cells (Ostrov et al. 2021). Although this has not been assessed for PB28 in SARS-CoV-2-infected Vero-E6 or A549-ACE2 cells, we examined a potential mechanism-based mode of PB28 action by fitting a viral dynamics model to viral load data from PB28-treated A549-ACE2 cell infections. Because reduced cytopathic effects may result from decreased viral entry, we modeled PB28’s antiviral activity as acting on the infection rate, $\beta$, (Equations (S.1)–(S.5) in Online Resource 1). Fits of the viral dynamics model, assuming a PB28-induced reduction in the infection rate, to SARS-CoV-2 viral load data (Figure S14 in Online Resource 1) were statistically significantly worse (p-value $<10^{-5}$) than those assuming a PB28-induced reduction in the viral production rate (Figure 3). However, despite the worse fit, Figure S15 in Online Resource 1 shows substantial overlap in the log-likelihood distributions for both models, indicating that viral load data alone are insufficient to unambiguously resolve the underlying mechanism of PB28 action on SARS-CoV-2 replication.

The interpretation of our results is subject to several limitations. In particular, the duration of infectious phase, $\tau _\text {I}$, and the virus production rate constant, *p*, exhibited wide 95% credible intervals and could not be reliably estimated. This uncertainty propagated to the model predictions, yielding broad 95% credible bands for the cell population trajectories (Figure 4). A short infectious phase (small $\tau _\text {I}$), during which infected cells produce virus and thus generate a low lower credible bound, was compensated by a high virus production rate (large *p*). Because $\tau _\text {I}$ and *p* were strongly correlated, both parameters were sensitive to perturbations of their best-fit values (Figures S3 and S4 in Online Resource 1). Improved experimental resolution could enhance the practical identifiability of these parameters. The infection experiments were terminated at 97 h post-infection, when viral loads in the control condition were still increasing and had not yet reached a plateau, which likely impaired inference of $\tau _\text {I}$. In addition, limitations may arise from the model structure itself. As shown by Liyanage et al. (2024), the virus production parameter *p* is not structurally identifiable in the target-cell-limited viral dynamics model. These practical identifiability issues could be mitigated by incorporating more informative data, for example by performing additional infections at low multiplicity of infection to better resolve the early phase of viral accumulation.

In this study, we combined the time-resolved *in vitro* SARS-CoV-2 infection data with concentration-response end-point assays to jointly estimate viral life-cycle parameters and the properties of PB28. Our analysis revealed that $\text {IC}_{50}$ values depended on the modeling approach, with estimates from time-resolved infection data approximately twofold higher than those derived from end-point assays. We further demonstrated that $\text {IC}_{50}$ can vary depending on the time point at which the end-point infection is measured. These findings highlight the value of integrating dynamic modeling with minimal experimental data as a rational strategy to improve the interpretability of drug screens and to guide the mechanistic development of host-directed therapeutics.

## Supplementary Information

Below is the link to the electronic supplementary material.

Supplementary file 1 (pdf 3061 KB)

## Declarations

### Data availability

The experimental data presented in Figure 1 and all Python files and metadata used to generate the figures are available for download at https://github.com/VBernhauer/PB28-inhibition-SARS-CoV-2-model--Supplementary-Code.

## References

[CR1] Abate C, Niso M, Abatematteo FS, Contino M, Colabufo NA, Berardi F (2020) PB28, the sigma-1 and sigma-2 receptors modulator with potent anti-SARS-CoV-2 activity: A review about its pharmacological properties and structure affinity relationships. Front Pharmacol 11(589):810 33364961 10.3389/fphar.2020.589810 PMC7750835

[CR2] Abatematteo FS, Delre P, Mercurio I, Rezelj VV, Siliqi D, Beaucourt S, Lattanzi G, Colabufo NA, Leopoldo M, Saviano M et al (2023) A conformational rearrangement of the SARS-CoV-2 host protein sigma-1 is required for antiviral activity: insights from a combined in-silico/in-vitro approach. Sci Rep 13(1):12798 37550340 10.1038/s41598-023-39662-w PMC10406941

[CR3] Baccam P, Beauchemin C, Macken CA, Hayden FG, Perelson AS (2006) Kinetics of influenza A virus infection in humans. J Virol 80(15):7590–7599 16840338 10.1128/JVI.01623-05 PMC1563736

[CR4] Bakowski MA, Beutler N, Wolff KC, Kirkpatrick MG, Chen E, Nguyen TTH, Riva L, Shaabani N, Parren M, Ricketts J et al (2021) Drug repurposing screens identify chemical entities for the development of COVID-19 interventions. Nat Commun 12(1):3309 34083527 10.1038/s41467-021-23328-0 PMC8175350

[CR5] Beauchemin CA, Kim YI, Yu Q, Ciaramella G, DeVincenzo JP (2019) Uncovering critical properties of the human respiratory syncytial virus by combining in vitro assays and in silico analyses. PLoS ONE 14(4):e0214,708 10.1371/journal.pone.0214708 PMC6464176 30986239

[CR6] Bernhauerová V, Rezelj VV, Vignuzzi M (2020) Modelling degradation and replication kinetics of the Zika virus in vitro infection. Viruses 12(5):547 32429277 10.3390/v12050547 PMC7290367

[CR7] Bernhauerová V, Lisowski B, Rezelj VV, Vignuzzi M (2021) Mathematical modelling of SARS-CoV-2 infection of human and animal host cells reveals differences in the infection rates and delays in viral particle production by infected cells. J Theor Biol 531(110):895 10.1016/j.jtbi.2021.110895 PMC8418984 34499915

[CR8] Brimson JM, Prasanth MI, Malar DS, Brimson S, Thitilertdecha P, Tencomnao T (2021) Drugs that offer the potential to reduce hospitalization and mortality from SARS-CoV-2 infection: The possible role of the sigma-1 receptor and autophagy. Expert Opin Ther Targets 25(6):435–449 34236922 10.1080/14728222.2021.1952987 PMC8290373

[CR9] Chakraborty C, Sharma AR, Bhattacharya M, Agoramoorthy G, Lee SS (2021) The drug repurposing for COVID-19 clinical trials provide very effective therapeutic combinations: lessons learned from major clinical studies. Front Pharmacol 12(704):205 10.3389/fphar.2021.704205 PMC8636940 34867318

[CR10] Consortium WST (2021) Repurposed antiviral drugs for Covid-19-interim WHO solidarity trial results. N Engl J Med 384(6):497–511 10.1056/NEJMoa2023184 PMC7727327 33264556

[CR11] Drewes N, Fang X, Gupta N, Nie D (2025) Pharmacological and Pathological Implications of Sigma-1 Receptor in Neurodegenerative Diseases. Biomedicines 13(6):1409 40564128 10.3390/biomedicines13061409 PMC12190457

[CR12] Foreman-Mackey D, Hogg DW, Lang D, Goodman J (2013) emcee: the MCMC hammer. Publ Astron Soc Pac 125(925):306

[CR13] Goodman J, Weare J (2010) Ensemble samplers with affine invariance. Communications in Applied Mathematics and Computational Science 5(1):65–80 10.2140/camcos.2010.5.31 PMC3815654 24194691

[CR14] Gordon DE, Jang GM, Bouhaddou M, Xu J, Obernier K, White KM, O’Meara MJ, Rezelj VV, Guo JZ, Swaney DL et al (2020) A SARS-CoV-2 protein interaction map reveals targets for drug repurposing. Nature 583(7816):459–468 32353859 10.1038/s41586-020-2286-9 PMC7431030

[CR15] Guy RK, DiPaola RS, Romanelli F, Dutch RE (2020) Rapid repurposing of drugs for COVID-19. Science 368(6493):829–830 32385101 10.1126/science.abb9332

[CR16] Han N, Hwang W, Tzelepis K, Schmerer P, Yankova E, MacMahon M, Lei W, M Katritsis N, Liu A, Felgenhauer U, et al (2021) Identification of SARS-CoV-2–induced pathways reveals drug repurposing strategies. Science Advances 7(27):eabh3032 10.1126/sciadv.abh3032 PMC8245040 34193418

[CR17] Heyer A, Günther T, Robitaille A, Lütgehetmann M, Addo MM, Jarczak D, Kluge S, Aepfelbacher M, Zur Wiesch JS, Fischer N, et al (2022) Remdesivir-induced emergence of SARS-CoV2 variants in patients with prolonged infection. Cell Reports Medicine 3(9) 10.1016/j.xcrm.2022.100735 PMC9378267 36075217

[CR18] Hurtado PJ, Kirosingh AS (2019) Generalizations of the ‘Linear Chain Trick’: incorporating more flexible dwell time distributions into mean field ODE models. J Math Biol 79(5):1831–1883 31410551 10.1007/s00285-019-01412-w PMC6800873

[CR19] Izzo NJ, Colom-Cadena M, Riad AA, Xu J, Singh M, Abate C, Cahill MA, Spires-Jones TL, Bowen WD, Mach RH, et al (2020) Proceedings from the fourth international symposium on -2 receptors: Role in health and disease. Eneuro 7(6) 10.1523/ENEURO.0317-20.2020 PMC7643771 33028631

[CR20] Kakizoe Y, Nakaoka S, Beauchemin CA, Morita S, Mori H, Igarashi T, Aihara K, Miura T, Iwami S (2015) A method to determine the duration of the eclipse phase for in vitro infection with a highly pathogenic SHIV strain. Sci Rep 5(1):10,371 10.1038/srep10371 PMC4440524 25996439

[CR21] Kaufmann SH, Dorhoi A, Hotchkiss RS, Bartenschlager R (2018) Host-directed therapies for bacterial and viral infections. Nat Rev Drug Discovery 17(1):35–56 28935918 10.1038/nrd.2017.162 PMC7097079

[CR22] Krippendorff BF, Neuhaus R, Lienau P, Reichel A, Huisinga W (2009) Mechanism-based inhibition: deriving KI and kinact directly from time-dependent IC50 values. SLAS Discovery 14(8):913–923 10.1177/1087057109336751 19675314

[CR23] Laporte M, Jochmans D, Bardiot D, Desmarets L, Debski-Antoniak OJ, Mizzon G, Abdelnabi R, Leyssen P, Chiu W, Zhang Z et al (2025) A coronavirus assembly inhibitor that targets the viral membrane protein. Nature 640(8058):514–523 40140569 10.1038/s41586-025-08773-x PMC11981944

[CR24] Li G, Hilgenfeld R, Whitley R, De Clercq E (2023) Therapeutic strategies for COVID-19: progress and lessons learned. Nat Rev Drug Discovery 22(6):449–475 37076602 10.1038/s41573-023-00672-y PMC10113999

[CR25] Liang Y, Quan X, Gu R, Meng Z, Gan H, Wu Z, Sun Y, Pan H, Han P, Liu S, et al (2024) Repurposing existing drugs for the treatment of COVID-19/SARS-CoV-2: A review of pharmacological effects and mechanism of action. Heliyon 10(16) 10.1016/j.heliyon.2024.e35988 PMC11379597 39247343

[CR26] Liyanage YR, Heitzman-Breen N, Tuncer N, Ciupe SM (2024) Identifiability investigation of within-host models of acute virus infection. Math Biosci Eng 21(10):7394–7420 39696868 10.3934/mbe.2024325 PMC12182237

[CR27] Markov PV, Ghafari M, Beer M, Lythgoe K, Simmonds P, Stilianakis NI, Katzourakis A (2023) The evolution of SARS-CoV-2. Nat Rev Microbiol 21(6):361–379 37020110 10.1038/s41579-023-00878-2

[CR28] Mslati H, Gentile F, Perez C, Cherkasov A (2021) Comprehensive consensus analysis of SARS-CoV-2 drug repurposing campaigns. J Chem Inf Model 61(8):3771–3788 34313439 10.1021/acs.jcim.1c00384

[CR29] Murphy H, McCarthy G, Dobrovolny HM (2020) Understanding the effect of measurement time on drug characterization. PLoS ONE 15(5):e0233,031 10.1371/journal.pone.0233031 PMC7224495 32407356

[CR30] Ostrov DA, Bluhm AP, Li D, Khan JQ, Rohamare M, Rajamanickam K, Bhanumathy K, K, Lew J, Falzarano D, Vizeacoumar FJ, et al (2021) Highly specific sigma receptor ligands exhibit anti-viral properties in SARS-CoV-2 infected cells. Pathogens 10(11):1514 10.3390/pathogens10111514 PMC8620039 34832669

[CR31] Owen DR, Allerton CM, Anderson AS, Aschenbrenner L, Avery M, Berritt S, Boras B, Cardin RD, Carlo A, Coffman KJ et al (2021) An oral SARS-CoV-2 Mpro inhibitor clinical candidate for the treatment of COVID-19. Science 374(6575):1586–1593 34726479 10.1126/science.abl4784

[CR32] Paradis EG, Pinilla LT, Holder BP, Abed Y, Boivin G, Beauchemin CA (2015) Impact of the H275Y and I223V mutations in the neuraminidase of the 2009 pandemic influenza virus in vitro and evaluating experimental reproducibility. PLoS ONE 10(5):e0126,115 10.1371/journal.pone.0126115 PMC4439092 25992792

[CR33] Pinilla LT, Holder BP, Abed Y, Boivin G, Beauchemin CA (2012) The H275Y neuraminidase mutation of the pandemic A/H1N1 influenza virus lengthens the eclipse phase and reduces viral output of infected cells, potentially compromising fitness in ferrets. J Virol 86(19):10651–10660 22837199 10.1128/JVI.07244-11 PMC3457267

[CR34] Sánchez-Díez M, Romero-Jiménez P, Alegría-Aravena N, Gavira-O’Neill C, Vicente-García E, Quiroz-Troncoso J, González-Martos R, Ramírez-Castillejo C, Pastor J (2025) Assessment of cell viability in drug therapy: IC50 and other new time-independent indices for evaluating chemotherapy efficacy. Pharmaceutics 17(2):247 40006615 10.3390/pharmaceutics17020247 PMC11859577

[CR35] Sauvat A, Ciccosanti F, Colavita F, Di Rienzo M, Castilletti C, Capobianchi MR, Kepp O, Zitvogel L, Fimia GM, Piacentini M et al (2020) On-target versus off-target effects of drugs inhibiting the replication of SARS-CoV-2. Cell Death & Disease 11(8):656 32814759 10.1038/s41419-020-02842-x PMC7434849

[CR36] Schreiber A, Ludwig S (2025) Host-targeted antivirals against SARS-CoV-2 in clinical development-Prospect or disappointment? Antiviral Res 235(106):101 10.1016/j.antiviral.2025.106101 39923941

[CR37] Singh M, Shanmukha S, Eldesouki RE, Harraz MM (2025) FDA-approved drug repurposing screen identifies inhibitors of SARS-CoV-2 pseudovirus entry. Front Pharmacol 16(1537):912 10.3389/fphar.2025.1537912 PMC11955658 40166473

[CR38] Song G, Cheng L, Li D, Cheng J, Chao S, Li X, Zhu R, Zhang C, Li J (2025) Broad-spectrum antiviral activity of the sigma-1 receptor antagonist PB28 against coronaviruses. Front Microbiol 16(1636):035 10.3389/fmicb.2025.1636035 PMC12378744 40873706

[CR39] Staroverov V, Nersisyan S, Galatenko A, Alekseev D, Lukashevich S, Polyakov F, Anisimov N, Tonevitsky A (2023) Development of a novel mathematical model that explains SARS-CoV-2 infection dynamics in Caco-2 cells. PeerJ 11(e14):828 10.7717/peerj.14828 PMC9899056 36748087

[CR40] Staroverov V, Galatenko A, Knyazev E, Tonevitsky A (2024) Mathematical model explains differences in Omicron and Delta SARS-CoV-2 dynamics in Caco-2 and Calu-3 cells. PeerJ 12(e16):964 10.7717/peerj.16964 PMC10981414 38560455

[CR41] Teoh SL, Lim YH, Lai NM, Lee SW (2020) Directly acting antivirals for COVID-19: Where do we stand? Front Microbiol 11:1857 32849448 10.3389/fmicb.2020.01857 PMC7419656

[CR42] Utama R, Hapsari R, Puspitasari I, Sari D, Hendrianingtyas M, Nurainy N (2022) Self-collected gargle specimen as a patient-friendly sample collection method for COVID-19 diagnosis in a population context. Sci Rep 12(1):3706 35260654 10.1038/s41598-022-07690-7 PMC8904449

[CR43] Warren TK, Jordan R, Lo MK, Ray AS, Mackman RL, Soloveva V, Siegel D, Perron M, Bannister R, Hui HC et al (2016) Therapeutic efficacy of the small molecule GS-5734 against Ebola virus in rhesus monkeys. Nature 531(7594):381–385 26934220 10.1038/nature17180 PMC5551389

[CR44] Zhang C, Jiang Q, Liu Z, Li N, Hao Z, Song G, Li D, Chen M, Lin L, Liu Y et al (2024) SARS-CoV-2 NSP6 reduces autophagosome size and affects viral replication via sigma-1 receptor. J Virol 98(11):e00,754-24 10.1128/jvi.00754-24 PMC11575221 39445785

[CR45] Zitzmann C, Schmid B, Ruggieri A, Perelson AS, Binder M, Bartenschlager R, Kaderali L (2020) A coupled mathematical model of the intracellular replication of dengue virus and the host cell immune response to infection. Front Microbiol 11:725 32411105 10.3389/fmicb.2020.00725 PMC7200986

