# Supplementary material for: In Vitro Analysis and Dynamic Modeling of SARS-CoV-2 Infection Inhibition by Sigma-1 Receptor Antagonist PB28
Source: Bull Math Biol. 2026 May 23;88(6):96. doi: 10.1007/s11538-026-01642-2 (PMC13198474; doi:10.1007/s11538-026-01642-2)
Supplement: Supplementary file 1 — (pdf 3061 KB) [file 11538_2026_1642_MOESM1_ESM.pdf]

In vitro analysis and dynamic modeling of SARS-CoV-2 infection inhibition by sigma-1 receptor antagonist PB28

Supplementary Information

Bartek Lisowski[1], Veronica V. Rezelj[2], Marco Vignuzzi[3,4,5], Carmen Abate[6], and Veronika Bernhauerová[7✉]

[1]Chair of Pharmaceutical Technology and Biopharmaceutics, Faculty of Pharmacy, Jagiellonian University Medical College, Kraków, Poland
[2]Institut Pasteur, Viral Populations and Pathogenesis Unit, Department of Virology, CNRS UMR 3569, F-75015 Paris, France
[3]A*STAR Infectious Diseases Labs (A*STAR ID Labs), Agency for Science, Technology and Research (A*STAR), 8A Biomedical Grove, Immunos #05-13, Singapore, 138648, Singapore
[4]Infectious Diseases Translational Research Programme, Department of Microbiology and Immunology, Yong Loo Lin School of Medicine, National University of Singapore, Singapore, Singapore
[5]Lee Kong Chian School of Medicine, Nanyang Technological University, Singapore, Singapore
[6]Department of Pharmacy-Pharmaceutical Sciences, University of Bari Aldo Moro, via E. Orabona, 4, 70125 Bari, Italy
[7]Department of Biophysics and Physical Chemistry, Faculty of Pharmacy, Charles University, Heyrovského 1203, 500 03 Hradec Králové, Czech Republic
[✉]Authors for correspondence: bernhauve@faf.cuni.cz

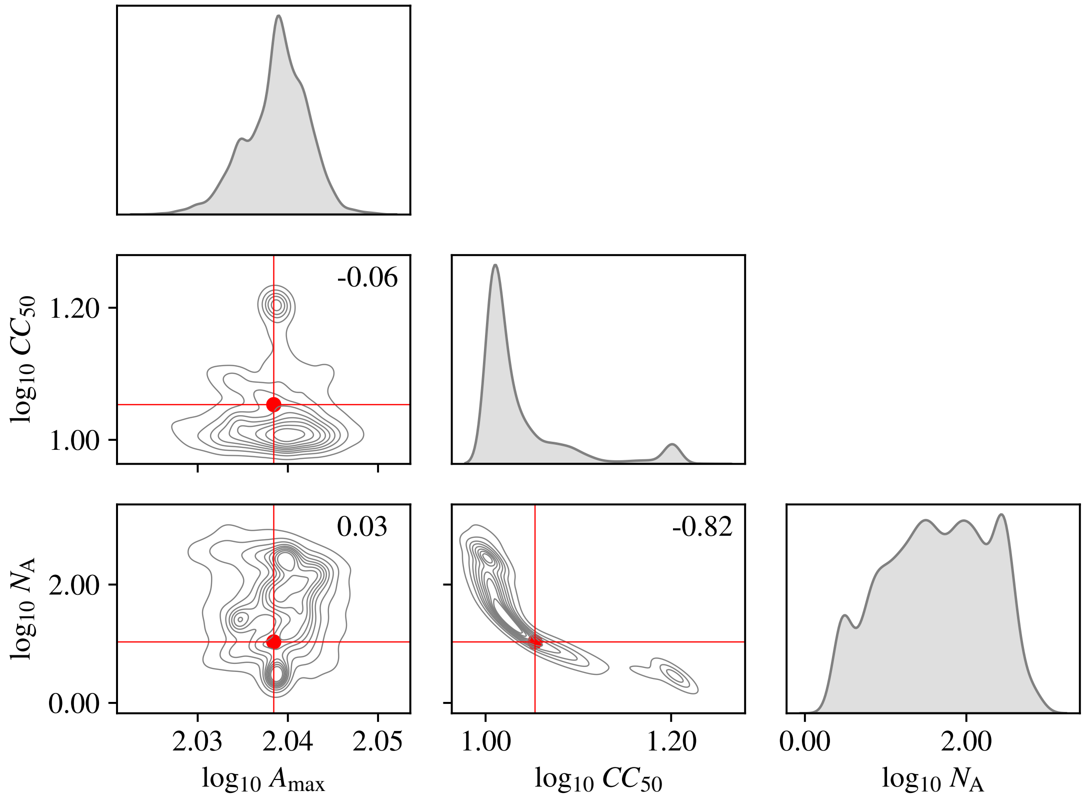

Figure S1. PB28 parameters influencing A549-ACE2 cell viability. Equation (13) in the main text was fitted to the relative percentages of viable cells (relative to the control) using MCMC (details are in Materials and Methods in the main text). A total of 6 independent chains were run for 20,000 steps, with a burn-in of 10,000 steps. Thinning was applied by accepting every 10th step. A total of 6,000 accepted parameter sets were considered. The value in the upper-right corner represents the Pearson correlation coefficient for the corresponding parameter pair. The best-fit parameter value (Table 2 in the main text) is denoted by a red point.

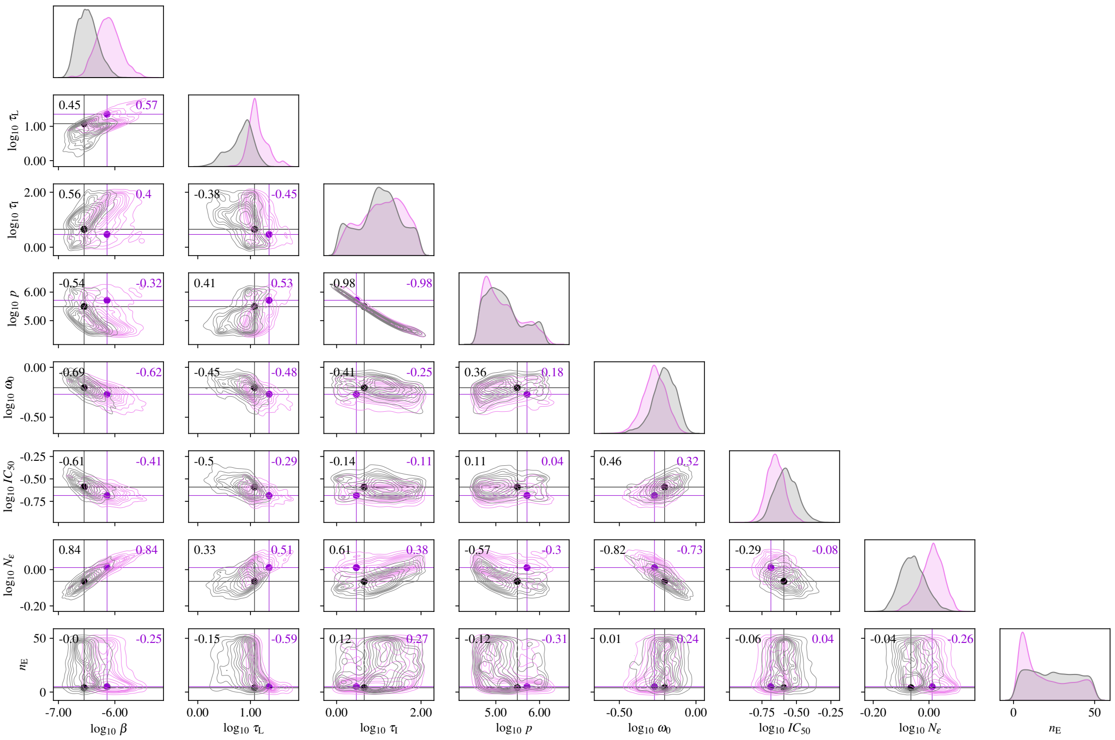

Figure S2. SARS-CoV-2 infection parameters for time-resolved experimental infection scheme. Posterior histograms and pair-wise parameter plots were obtained from simultaneously fitting of viral dynamics model (Equations (1)–(5) in the main text) to viral load data obtained from either only time-resolved (pink, Figure S12G–I) or both, end-point and time-resolved (grey, Figures 3A–F and 3G–I in the main text, respectively) experimental infection schemes using MCMC (details are in Materials and Methods in the main text). A total of 16 independent chains were run for 20,000 steps, with a burn-in of 10,000 steps. Thinning was applied by accepting every 10th step. A total of 16,000 accepted parameter sets were considered. The values in the upper-left and upper-right corners represent the Pearson correlation coefficients for the corresponding parameter pair. The best-fit parameter values (Table 1 in the main text) are denoted by filled points.

Local sensitivity analysis

To quantify the impact of parameter variability on the behavior of the viral dynamics model (Equations (1)–(5) in the main text), we conducted a one-at-a-time sensitivity analysis. The parameters examined were the infection rate constant, β, the latent-phase duration, τ_L, the infectious-phase duration, τ_I, the number of latent compartments, $n_{\rm L}$, the viral production rate constant, p, the parameter governing the washing process, ω_0, the half-maximal inhibitory concentration, $IC_{50,\epsilon}$, and the Hill coefficient describing cooperativity in the PB28 effect, N_ϵ. Each parameter was perturbed independently by $\pm 50\%$ relative to its maximum-likelihood (best-fit) estimate, while all remaining parameters were held fixed.

The parameters most sensitive to perturbations in their best-fit values were $\tau_{\rm I}$ (Figure S3), p (Figure S4), β (Figure S5), and $\tau_{\rm L}$ (Figure S6) . Increases (decreases) in $\tau_{\rm I}$, p, and β led to corresponding increases (decreases) in viral load. Specifically, increasing β raised the proportion of infected cells and, consequently, virus production. Similarly, increases (decreases) in $\tau_{\rm I}$ allowed infected cells to produce virus for longer (shorter) periods, resulting in greater (lower) viral accumulation. In contrast, increases (decreases) in $\tau_{\rm L}$ produced decreases (increases) in viral load, reflecting the effect of a prolonged (shortened) delay in virus production.

Variations in ω_0 did not significantly affect viral load (Figure S7), and the parameter $n_{\rm L}$ was not sensitive to perturbations in its best-fit value (Figure S8).

Sensitivity of parameters characterizing PB28 inhibition efficacy, $IC_{50,\epsilon}$ and N_ϵ, to perturbations in their best-fit values varied with PB28 concentration (Figures S9 and S10). Increases (decreases) in $IC_{50,\epsilon}$, and thus decreases (increases) in the PB28 inhibition efficacy, ϵ, resulted in increased (decreased) viral load. Increases (decreases) in N_ϵ resulted in increases (decreases) in viral load when the PB28 concentration was lower than $IC_{50,\epsilon}$. Indeed, if $IC_{50,\epsilon} > C$ (i.e., $\frac{IC_{50,\epsilon}}{C} > 1$) and assuming $\epsilon_{\rm max} = 1$, then for any $N_\epsilon^* > N_\epsilon$, it follows that, it follows that

$$\epsilon^* = \frac{1}{\left(\frac{IC_{50,\epsilon}}{C}\right)^{N_\epsilon^*} + 1} < \epsilon = \frac{1}{\left(\frac{IC_{50,\epsilon}}{C}\right)^{N_\epsilon} + 1},$$

resulting in lower inhibition efficacy and higher viral load. Similarly, increases (decreases) in N_ϵ resulted in corresponding decreases (increases) in viral load when the PB28 concentration, C, exceeded $IC_{50,\epsilon}$.

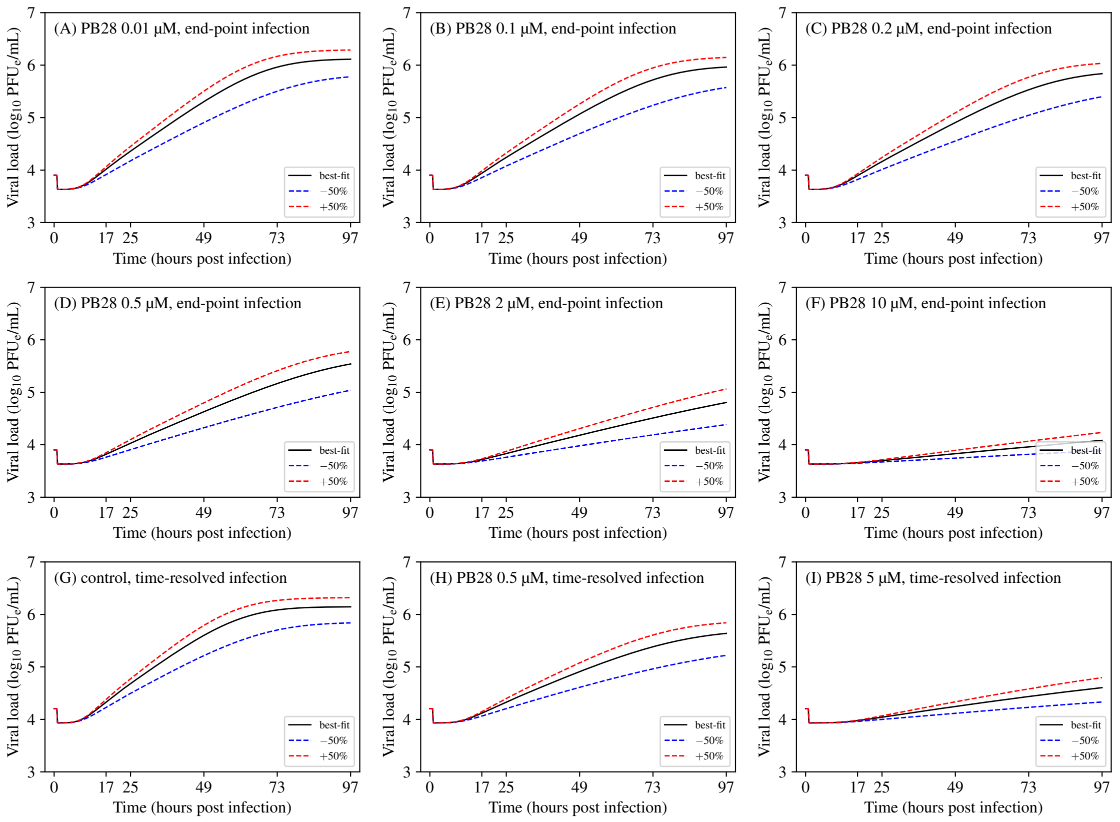

Figure S3. Local sensitivity analysis of infectious phase duration, τ_I. The parameter τ_I was varied by 50% from the best-fit value (Table 1 in the main text), and the corresponding solutions of Equations (1)–(5) in the main text were displayed as follows: a black solid line was used for the solutions associated with the best-fit parameter values; a red dashed line was used for the solutions associated with perturbations of +50%; and a blue dashed line was used for the solutions associated with perturbations in the best-fit parameter values by −50%.

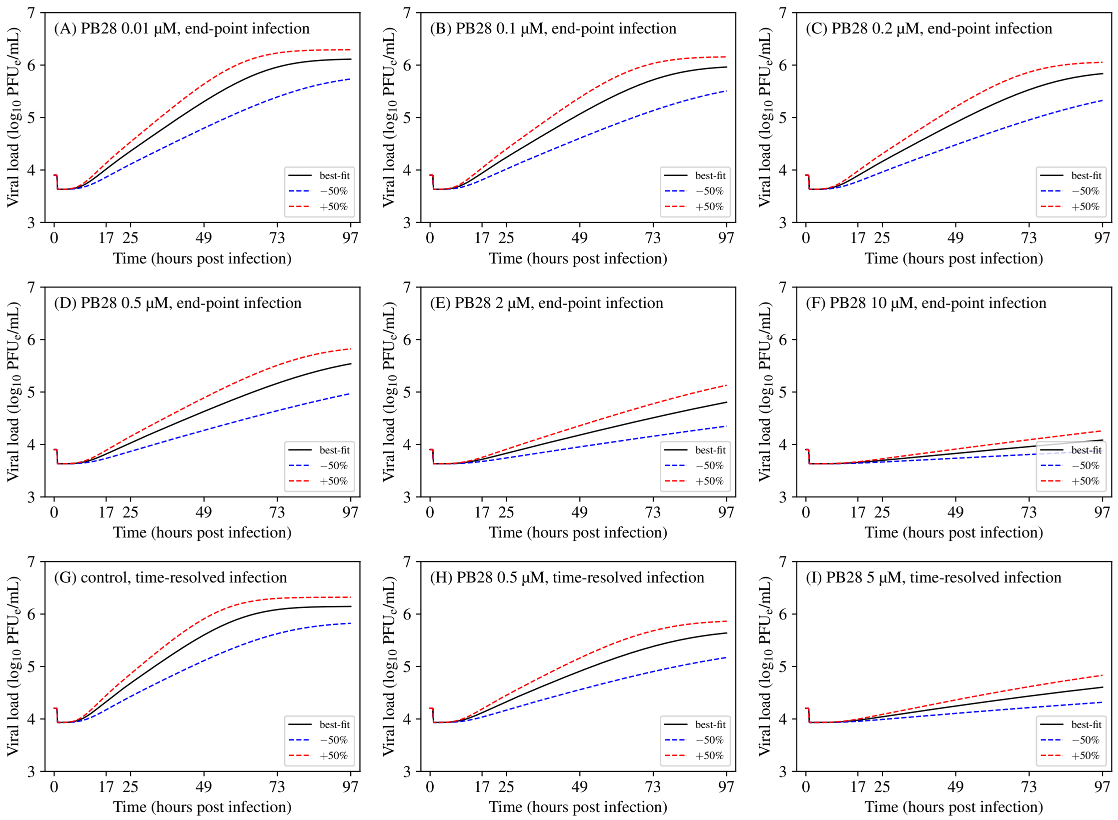

Figure S4. Local sensitivity analysis of virus production rate constant, p. The parameter p was varied by 50% from the best-fit value (Table 1 in the main text), and the corresponding solutions of Equations (1)–(5) in the main text were displayed as follows: a black solid line was used for the solutions associated with the best-fit parameter values; a red dashed line was used for the solutions associated with perturbations of +50%; and a blue dashed line was used for the solutions associated with perturbations in the best-fit parameter values by −50%.

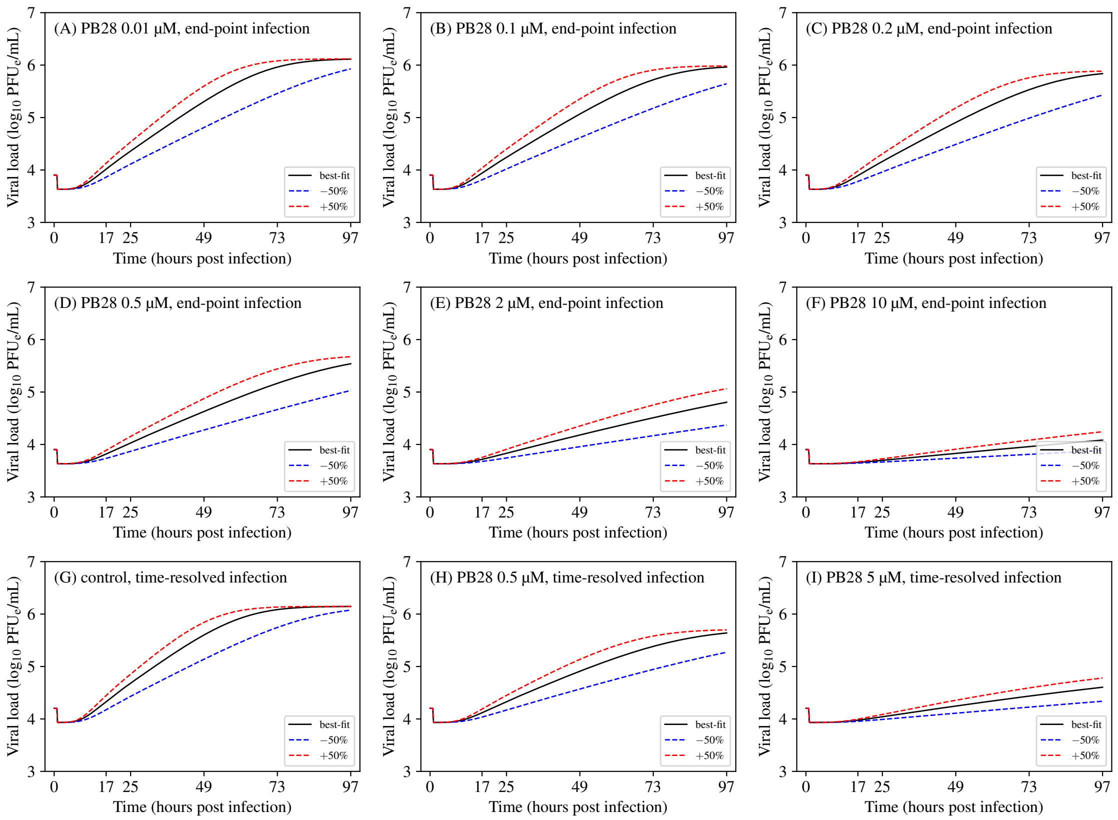

Figure S5. Local sensitivity analysis of the infection rate constant, β. The parameter β was varied by 50% from the best-fit value (Table 1 in the main text), and the corresponding solutions of Equations (1)–(5) in the main text were displayed as follows: a black solid line was used for the solutions associated with the best-fit parameter values; a red dashed line was used for the solutions associated with perturbations of +50%; and a blue dashed line was used for the solutions associated with perturbations in the best-fit parameter values by −50%.

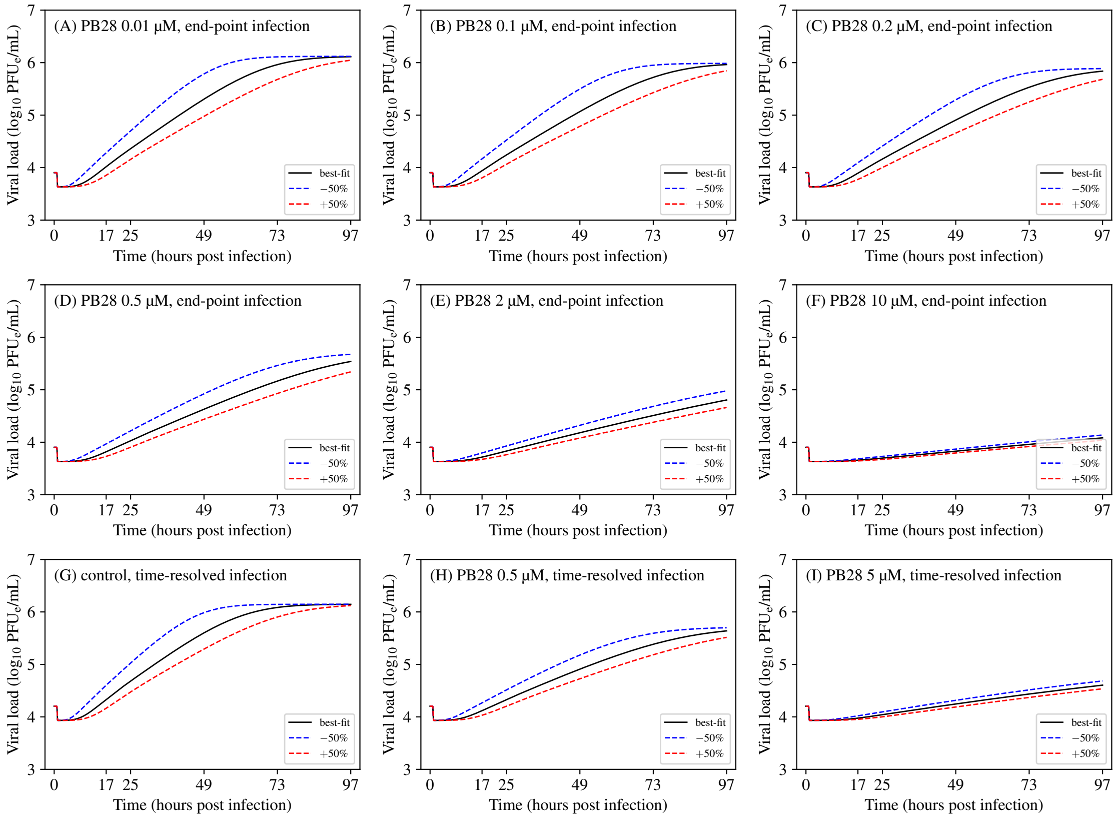

Figure S6. Local sensitivity analysis of latent phase duration, τ_L. The parameter τ_L was varied by 50% from the best-fit value (Table 1 in the main text), and the corresponding solutions of Equations (1)–(5) in the main text were displayed as follows: a black solid line was used for the solutions associated with the best-fit parameter values; a red dashed line was used for the solutions associated with perturbations of +50%; and a blue dashed line was used for the solutions associated with perturbations in the best-fit parameter values by −50%.

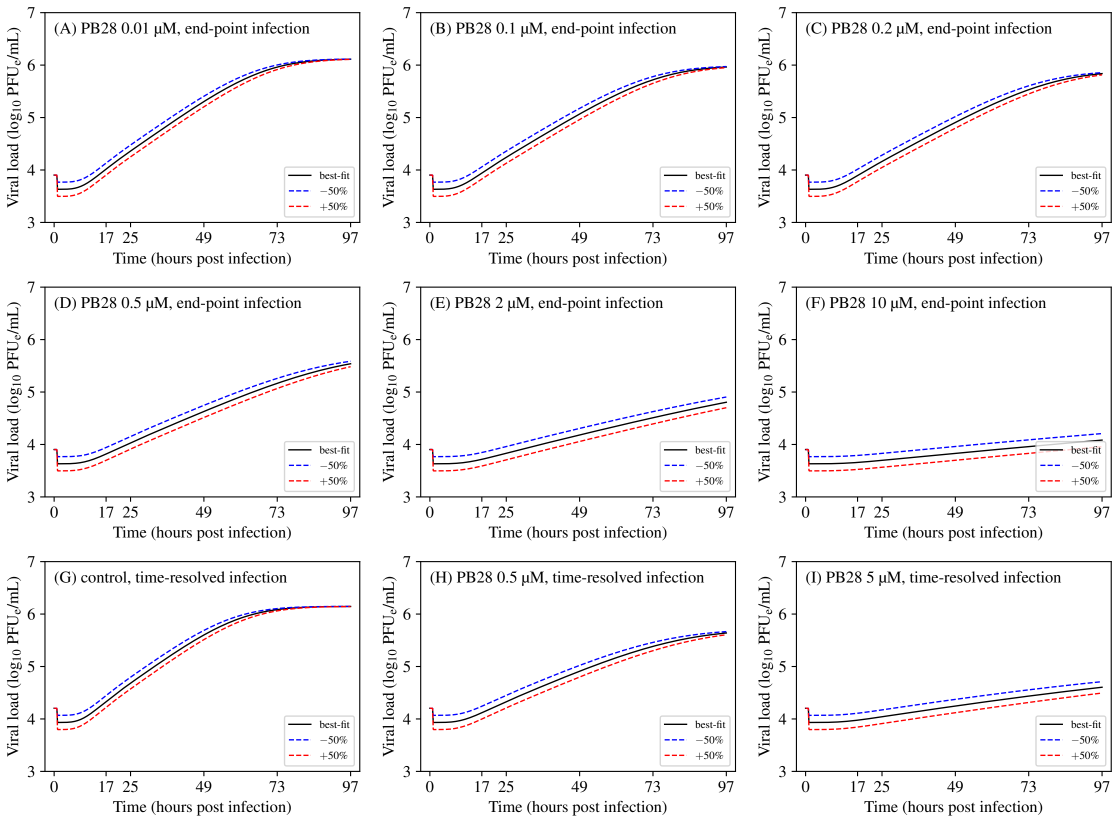

Figure S7. Local sensitivity analysis of washing rate constant, ω_0. The parameter ω_0 was varied by 50% from the best-fit value (Table 1 in the main text), and the corresponding solutions of Equations (1)–(5) in the main text were displayed as follows: a black solid line was used for the solutions associated with the best-fit parameter values; a red dashed line was used for the solutions associated with perturbations of +50%; and a blue dashed line was used for the solutions associated with perturbations in the best-fit parameter values by −50%.

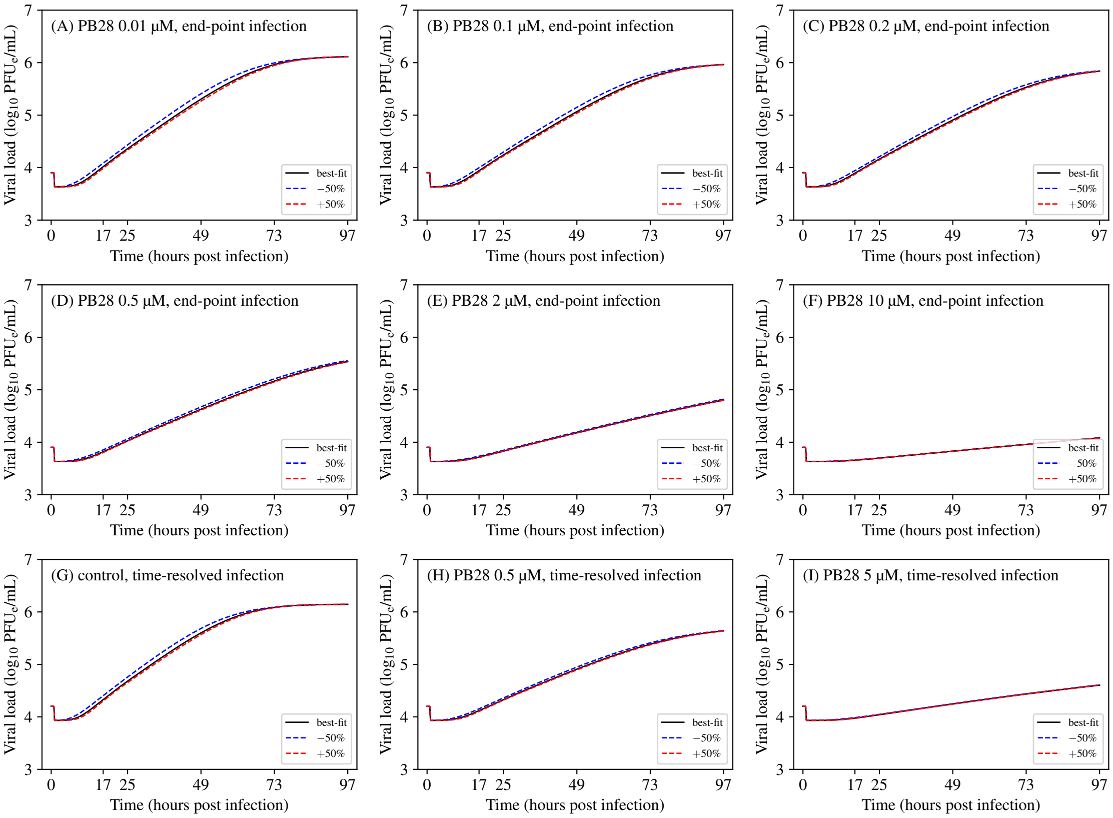

Figure S8. Local sensitivity analysis of number of latent phase compartments, $n_\mathbf{L}$. The parameter n_L was varied by 50% from the best-fit value (Table 1 in the main text), and the corresponding solutions of Equations (1)–(5) in the main text were displayed as follows: a black solid line was used for the solutions associated with the best-fit parameter values; a red dashed line was used for the solutions associated with perturbations of +50%; and a blue dashed line was used for the solutions associated with perturbations in the best-fit parameter values by −50%.

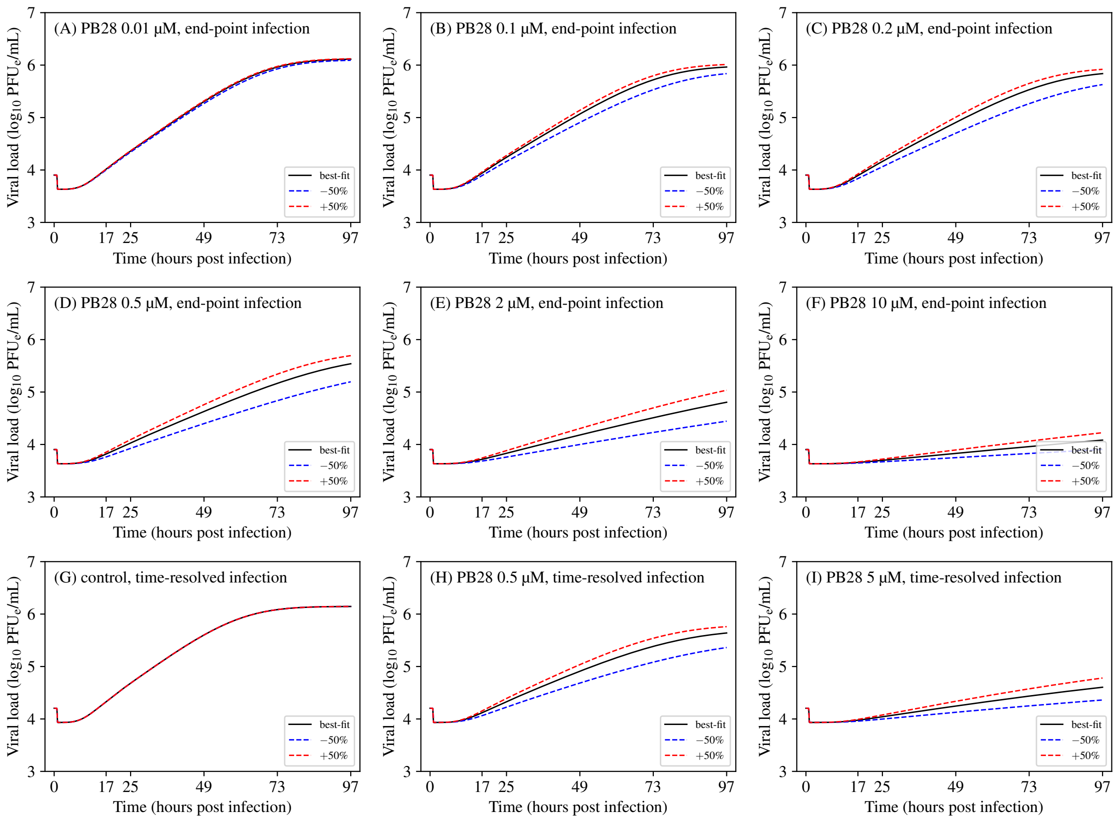

Figure S9. Local sensitivity analysis of PB28 half-maximum inhibition constant, IC_{50}. The parameter IC_{50} was varied by 50% from the best-fit value (Table 1 in the main text), and the corresponding solutions of Equations (1)–(5) in the main text were displayed as follows: a black solid line was used for the solutions associated with the best-fit parameter values; a red dashed line was used for the solutions associated with perturbations of +50%; and a blue dashed line was used for the solutions associated with perturbations in the best-fit parameter values by −50%.

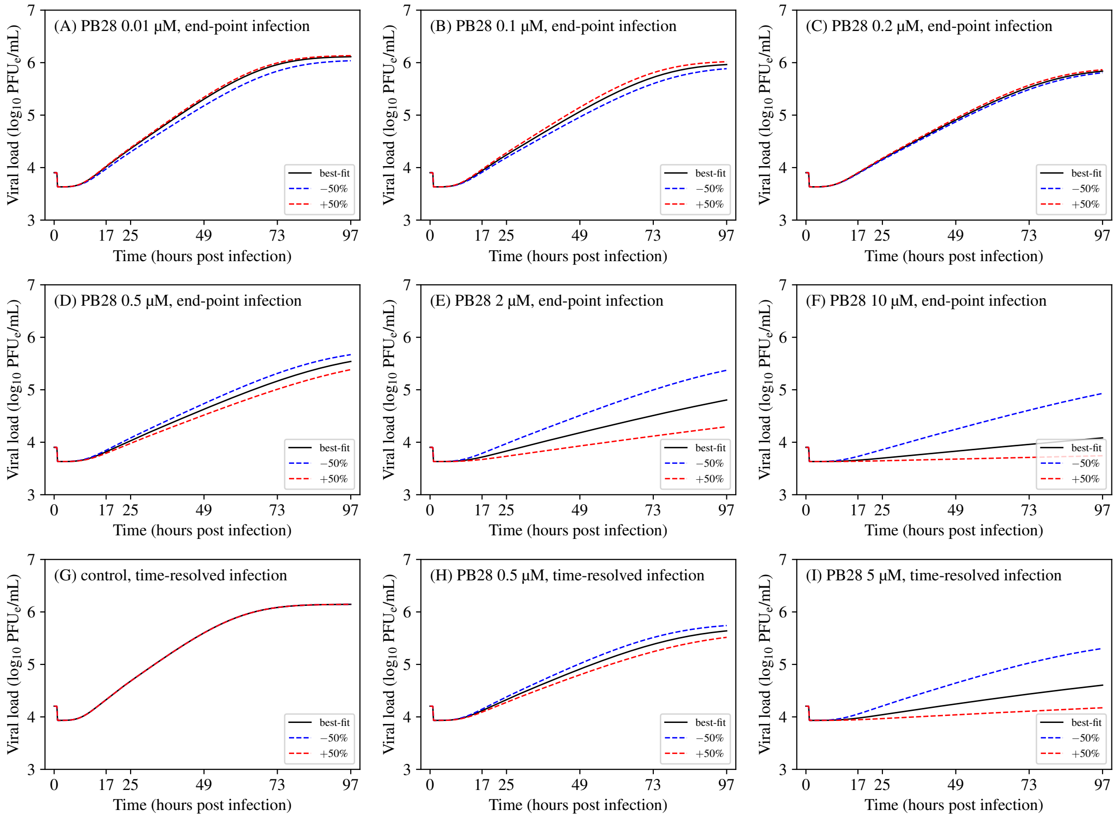

Figure S10. Local sensitivity analysis of Hill coeficient, N_ϵ. The parameter N_ϵ was varied by 50% from the best-fit value (Table 1 in the main text), and the corresponding solutions of Equations (1)–(5) in the main text were displayed as follows: a black solid line was used for the solutions associated with the best-fit parameter values; a red dashed line was used for the solutions associated with perturbations of +50%; and a blue dashed line was used for the solutions associated with perturbations in the best-fit parameter values by −50%.

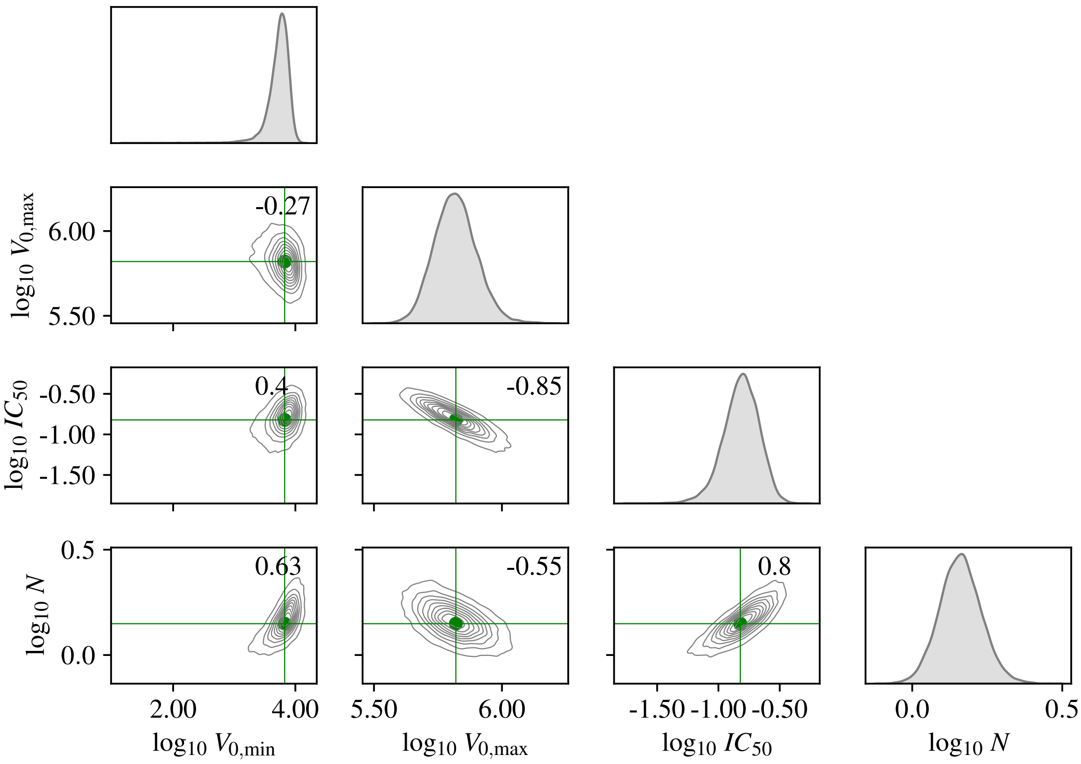

Figure S11. PB28 parameters influencing SARS-CoV-2 infection dynamics in A549-ACE2 cells. Equation (11) in the main text was fitted to SARS-CoV-2 viral loads using MCMC (details are in Materials and Methods in the main text). A total of 8 independent chains were run for 20,000 steps, with a burn-in of 10,000 steps. Thinning was applied by accepting every 5th step. A total of 16,000 accepted parameter sets were considered. The value in the upper-right corner represents the Pearson correlation coefficient for the corresponding parameter pair. The best-fit parameter value (Table 3 in the main text) is denoted by a green point.

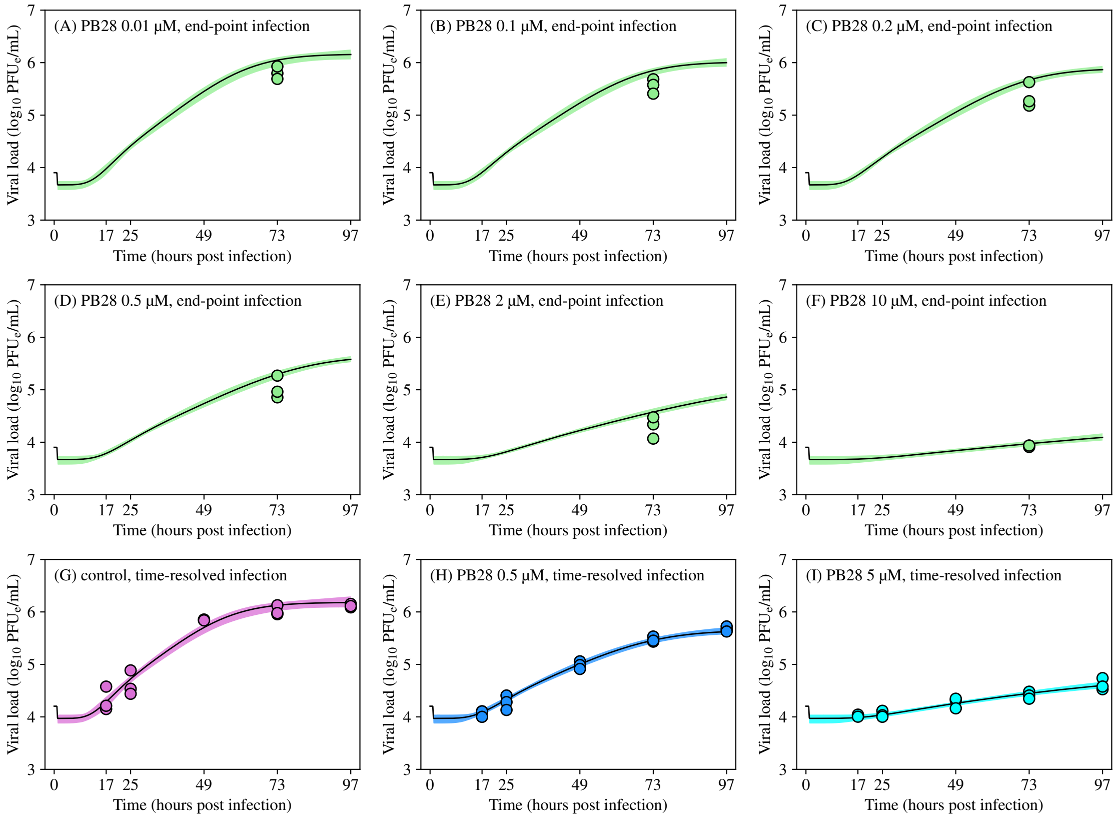

Figure S12. Fits of the viral dynamics model to SARS-CoV-2 viral load data. Equations (1)–(5) in the main text were fitted exclusively to SARS-CoV-2 viral loads obtained from time-resolved experimental infections (panels (G)–(I)) using MCMC (details are in Materials and Methods in the main text). A total of 16 independent chains were run for 20,000 steps, with a burn-in of 10,000 steps. Thinning was applied by accepting every 10th step. A total of 16,000 accepted parameter sets were considered. The maximum likelihood (best-fit) solutions are displayed as black solid lines. The 95% credible bands are displayed as filled areas. Legend as in Figure 1 in the main text.

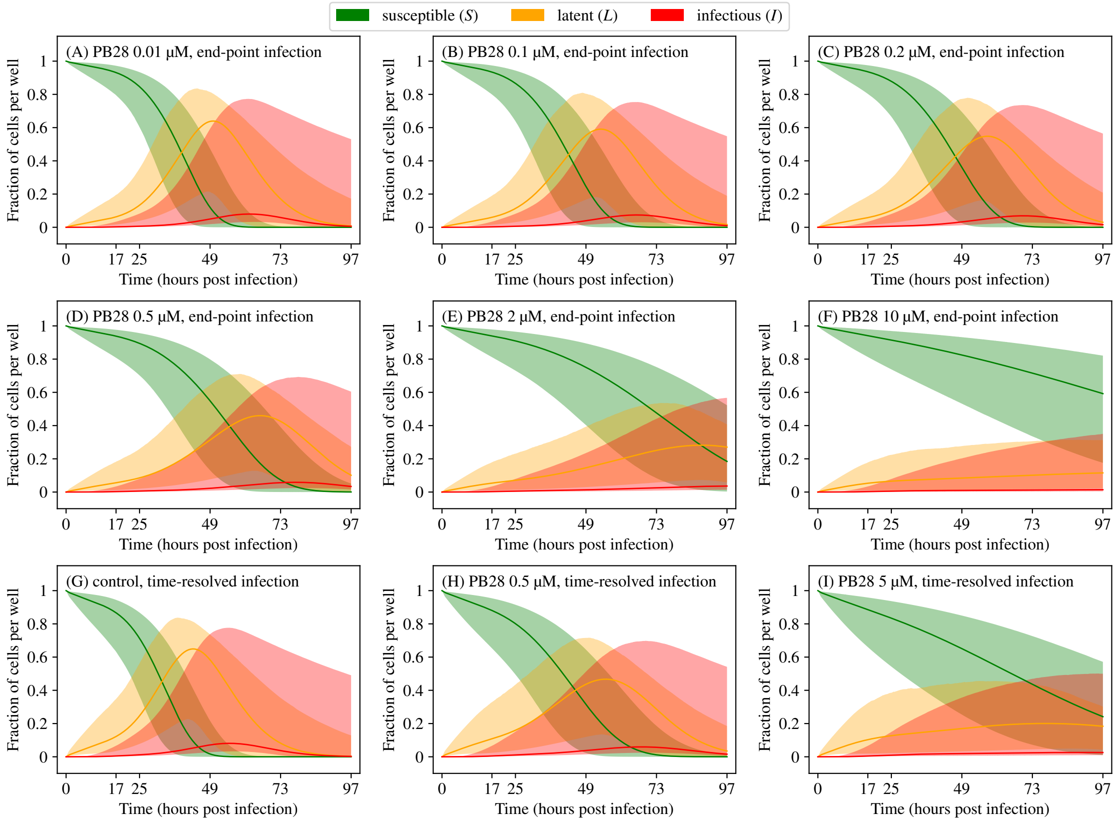

Figure S13. A549-ACE2 cell dynamics. Proportions of cells associated with fits of Equations (1)–(5) in the main text to SARS-CoV-2 viral loads obtained from time-resolved experimental infections (Figure S12G–I). Proportions of cells were calculated using the MCMC-accepted parameters, as described in Figure S12. The maximum likelihood (best-fit) solutions are displayed as solid lines. The 95% credible bands are displayed as filled areas.

PB28-induced inhibition of SARS-CoV-2 entry into A549-ACE2 cells

To explore how PB28-induced inhibition of SARS-CoV-2 entry into A549-ACE2 cells affects viral dynamics, we modified the viral dynamics model (Equations (1)–(5) in the main text) such that the antiviral effect of PB28 acts on the infection rate rather than on the virus production rate:

$$\frac{dS}{dt} = -(1-\epsilon)\,\beta\,V(t)\,S(t), \tag{S.1}$$

$$\frac{dL_1}{dt} = (1-\epsilon)\,\beta\,V(t)\,S(t) - \frac{n_{\mathrm{L}}}{\tau_{\mathrm{L}}}\,L_1(t) \tag{S.2}$$

$$\frac{dL_i}{dt} = \frac{n_{\mathrm{L}}}{\tau_{\mathrm{L}}}\,(L_{i-1}(t) - L_i(t)) \text{ for } i = 2,\ldots,n_{\mathrm{L}}, \tag{S.3}$$

$$\frac{dI}{dt} = \frac{n_{\mathrm{L}}}{\tau_{\mathrm{L}}}\,L_{n_{\mathrm{L}}}(t) - \frac{1}{\tau_{\mathrm{I}}}\,I(t), \tag{S.4}$$

$$\frac{dV}{dt} = p\,I(t) - \omega(t)\,V(t). \tag{S.5}$$

We assume that PB28 reduces the rate at which the virus infects cells by a factor $(1-\epsilon)$, where ϵ can vary between 0 (no reduction of viral entry into the cells) and 1 (complete inhibition of virus entry into the cells). The efficacy, ϵ, of PB28 in inhibiting the viral infection in Equations (S.1)–(S.2) is again modeled using Equation (6) in the main text.

We simultaneously fitted Equations (S.1)–(S.5) to viral load data obtained from end-point and time-resolved infections using the Markov chain Monte Carlo (MCMC) approach (details are in Materials and Methods in the main text). The parameters estimated were the infection rate constant, β; latent phase duration, τ_L; infectious phase duration, τ_I; the number of compartments for the latent phase, n_{L}; the virus production rate constant, p; the parameter characterizing the washing process, ω_0; PB28 half-maximum inhibition constant, $IC_{50,\epsilon}$; and Hill coefficient, N_ϵ. Parameter values and their 95% CIs are given in Table S1. Fits of Equations (S.1)–(S.5)) to viral load data are shown in Figure S14.

Table S1. SARS-CoV-2 and PB28 parameter estimates obtained from infection of A549-ACE2 cells (Equations (S.1)–(S.5)).

Parameter	Description	Units	ML value	95% CI
β	infection rate constant	mL/(PFU$_e$×h)	6.66×10^{-7}	$[2.38, 38.69]\times10^{-7}$
τ_L	duration of latent phase	h	11.49	[4.80, 30.71]
τ_I	duration of infectious phase	h	9.28	[1.14, 82.16]
n_L	number of latent phase compartments	-	20	[3, 48]
p	virus production rate constant	PFU$_e$/(h×mL)	1.1×10^5	$[0.271, 9.34]\times10^5$
ω_0	washing rate constant	-	0.48	[0.27, 0.67]
$\epsilon_{\max}$	maximum inhibition efficacy	-	1	fixed
$IC_{50,\epsilon}$	PB28 half-maximum inhibition constant	µM	0.11	[0.06, 0.23]
N_ϵ	Hill coeficient	-	0.86	[0.74, 1.11]

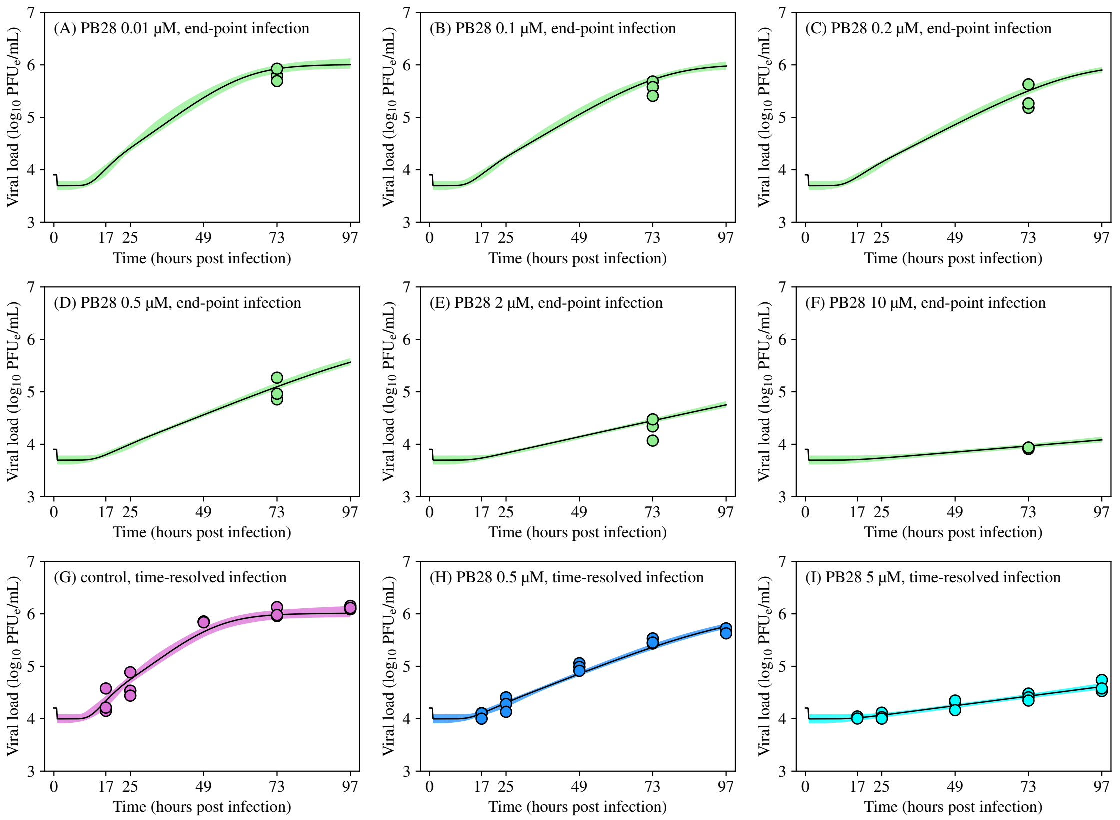

Figure S14. Fits of the viral dynamics model to SARS-CoV-2 viral load data assuming PB28-induced reduction of infection rate. Equations (S.1)–(S.5) were simultaneously fitted to SARS-CoV-2 viral loads obtained from end-point (panels A–F) and time-resolved (panels G–I) experimental infections using MCMC (details are in Materials and Methods). A total of 16 independent chains were run for 20,000 steps, with a burn-in of 10,000 steps. Thinning was applied by accepting every 10th step. A total of 16,000 accepted parameter sets were considered. The maximum likelihood (best-fit) solutions are displayed as black solid lines. The 95% credible bands are displayed as filled areas. Legend as in Figure 1 in the main text.

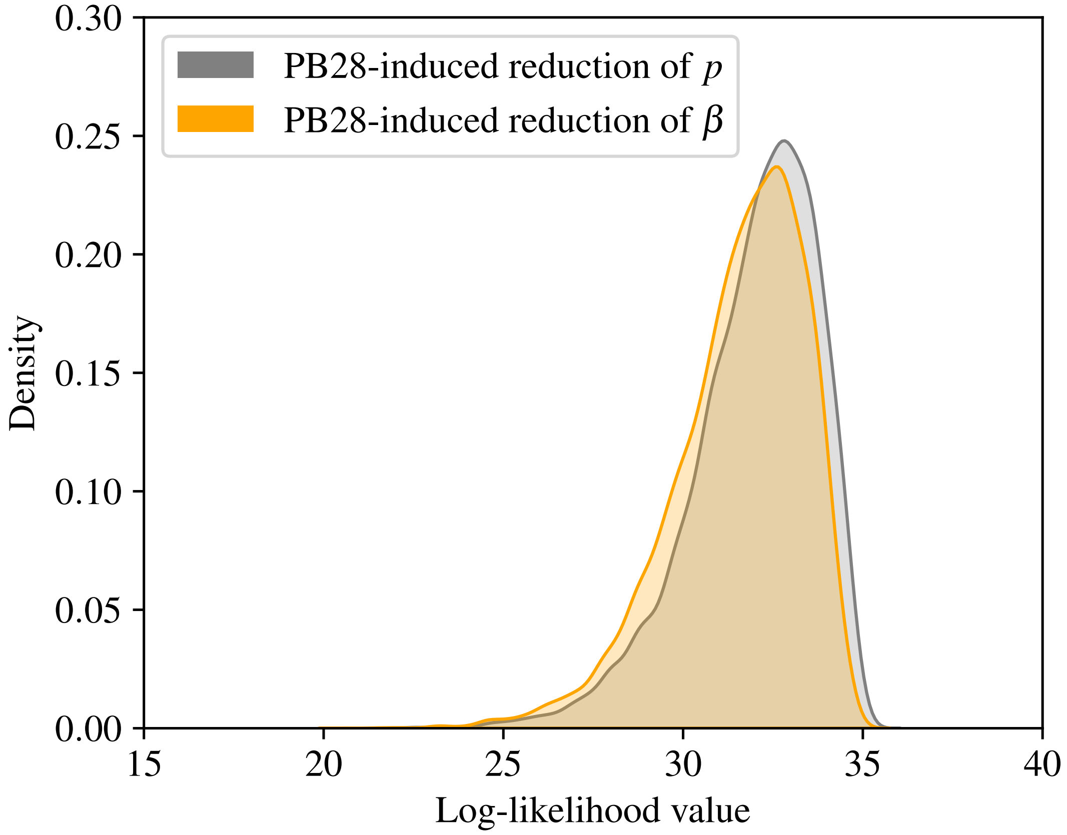

Figure S15. Goodness of fit of the viral dynamics model assuming PB28-induced reduction in virus production or the infection rate. Distributions of log-likelihood values (Equation (8) in the main text) resulting from fitting the viral dynamics model with PB28-induced reduction in virus production rate p (depicted in grey, Equations (1)–(5) in the main text) and with PB28-induced reduction of viral infection rate β (depicted in orange, Equations (S.1)–(S.5)) to viral load data obtained from end-point and time-resolved experimental infection schemes (Figures 3 in the main text and S14, respectively) using MCMC (details are in Materials and Methods in the main text and in the captions of Figures 3 in the main text and S14, respectively). Higher values indicate a better fit.
